# The arginine–phenylalanine–amide neuropeptide receptor family: Physiological effects, drug development, and structural insights

**DOI:** 10.4103/NRR.NRR-D-24-01313

**Published:** 2025-09-03

**Authors:** Yiming Liu, Shirui Jiang, Zhangsong Wu, Chen Qiu, Qiaohui Li, Rui Wang, Xiaoyi Yan, Song Wu, Geng Chen, Yang Du

**Affiliations:** 1 Kobilka Institute of Innovative Drug Discovery, Shenzhen Key Laboratory of Steroid Drug Discovery and Development, School of Medicine, The Chinese University of Hong Kong, Shenzhen, Guangdong Province, China; 2 The Huanan Affiliated Hospital of Shenzhen University, Shenzhen University, Shenzhen, Guangdong Province, China; 3 Biological Science Research Center, Academy for Advanced Interdisciplinary Studies, Southwest University, Chongqing, China

**Keywords:** appetite regulation, arginine–phenylalanine–amide, drug discovery, G protein-coupled receptor, neuropeptides, nerve regeneration, pain modulation, structure–activity relationship

## Abstract

The arginine–phenylalanine–amide neuropeptide receptor family comprises a subclass within the G protein-coupled receptor superfamily with crucial roles in physiological regulation. These receptors recognize and bind neuropeptides with an arginine–phenylalanine–amide motif, thereby participating in a variety of biological processes such as energy metabolism, pain perception, and reproductive functions. In this review, we explore the physiological and pathological processes involving these receptors and delve into the structure-activity relationships of their ligand peptides, clarifying the key structural motifs within these neuropeptides that determine their biological activity, pharmacological potency, and receptor selectivity. Particular emphasis is placed on their roles in modulating nociception, regulating appetite, and maintaining reproductive health. Additionally, we discuss the therapeutic potential of structure-based drug design targeting these receptors based on existing cryo-electron microscopy structures. The available structural insights into ligand-binding pockets and G protein–receptor interaction interfaces provide a clear perspective and valuable complement to ligand optimization.

## Introduction

G protein-coupled receptors (GPCRs), the largest family of eukaryotic transmembrane proteins, are highly promising targets for the development of next-generation drugs given their involvement in a wide range of human physiological processes (Rosenbaum et al., 2009). The GPCR superfamily conventionally includes six classes (A–F). Among these, Class A receptors, also known as rhodopsin-like receptors, represent the largest family and the most frequently targeted for drug development. Approximately 70%–80% of all FDA-approved drugs targeting GPCRs focus on Class A receptors, constituting up to 34% of all GPCR-targeted medications (Wold et al., 2019). The broad spectrum of transmembrane proteins within this class is responsive to a wide array of ligands, including neuropeptides (Duan et al., 2022). Neuropeptides are small, peptide-based signaling molecules with a large range of physiological functions. In the nervous system, they can act as neurotransmitters or neuromodulators, influencing the activity of co-released neurotransmitters. Neuropeptides and their receptors are widely distributed throughout both the central and peripheral nervous systems (Hökfelt et al., 2000).

In this review, we focus on a diverse group of neuropeptides, arginine–phenylalanine–amide (RF-amide) peptides, and their target GPCRs. RF-amide peptides derive their name from a conserved C-terminal motif containing an arginine (R) and an amidated phenylalanine (F) (RF-NH_2_). Their varying N-termini confer on these neuropeptides distinct biological activities (Findeisen et al., 2011). The first RF-amide peptide to be identified was the cardioexcitatory tetrapeptide FMRFamide, which was isolated from clam ganglia (Raffa, 1988). Since then, numerous RF-amide peptides have been discovered in a wide range of organisms. In mammals, the RF-amide peptides identified to date are classified into five subgroups—neuropeptide FF (NPFF), gonadotropin-inhibitory hormone (GnIH), prolactin-releasing peptide (PrRP), pyroglutamylated RF-amide peptide (QRFP), and kisspeptin. The peptides of these subgroups participate in diverse physiological processes modulated by the nervous system through the following Class A GPCRs: NPFF receptor 1 (NPFFR1, GPR147), NPFF receptor 2 (NPFFR2, GPR74), PrRP receptor (GPR10), QRFP receptor (GPR103), and kisspeptin receptor (GPR54) (Quillet et al., 2016). They are involved in modulating functions such as stress responses (Takayanagi and Onaka, 2010; Mamgain et al., 2021; Kovács et al., 2024), pain perception (Yang et al., 2008; Zhang et al., 2018; Xu et al., 2020), food intake (Dockray, 2004; Takayanagi and Onaka, 2010; Cook et al., 2022), reproduction (Zeydabadi Nejad et al., 2017; Navarro, 2020; Mamgain et al., 2021), circadian rhythms (Lee et al., 2017; Conrad et al., 2022), and cardiovascular activities (Nichols et al., 2012; Czerwińska et al., 2021). However, studies have identified cross-interactions among these RF-amide peptides and their five above-mentioned receptors, implying that these peptides exhibit affinity for more than one receptor (Oishi et al., 2010; Karnošová et al., 2021; Yakovlev et al., 2022). This indicates that the mechanisms underlying these ligand-receptor interactions remain incompletely understood and warrant further exploration. Advancing the understanding of these mechanisms requires the use of specific structural analysis tools, such as electron microscopy.

Electron microscopy, and particularly cryo-electron microscopy (cryo-EM), has achieved substantial advances over the past few decades. The continuous improvement in cryo-EM technology has revolutionized structure determination, facilitating the rapid growth in the number of high-resolution membrane protein structures determined (Earl et al., 2017). Cryo-EM overcomes the difficulty associated with crystallizing GPCRs and provides more detailed information on the structures of GPCR–G protein complexes compared with crystallization (Shihoya et al., 2024). This technology has also contributed to the gradual elucidation of the structures of RF-amide peptide receptors coupled with their corresponding G proteins.

In this review, we systematically examine the role of the RF-amide family signaling system in various physiological processes and highlight recent advances in the investigation of RF-amide family members as promising drug targets. We also discuss the emerging role of cryo-EM as an important tool for RF-amide peptide-based drug research.

## Search Strategy

For this narrative review, a comprehensive literature search was conducted across multiple databases, including PubMed, Google Scholar, ScienceDirect, Protein Data Bank (PDB), ChEMBL, and PubChem, covering publications from 1980 to 2024. The search terms used, alone or in combination, included “RF-amide peptides,” “structure–activity relationship,” (SAR) “kisspeptin,” “NPFF,” “GPR10,” and “GPR103.” Only English-language publications were considered. Irrelevant or duplicate articles were excluded. The screening process involved an initial review of titles and abstracts, followed by full-text assessments to determine final eligibility for inclusion in the review.

## Physiological Profiles of Arginine–Phenylalanine–Amide Peptide Receptors

### NPFF receptors (GPR147 and GPR74)

#### Ligands, chromosomal localization, and distribution of NPFF receptors

**Figure 1:**
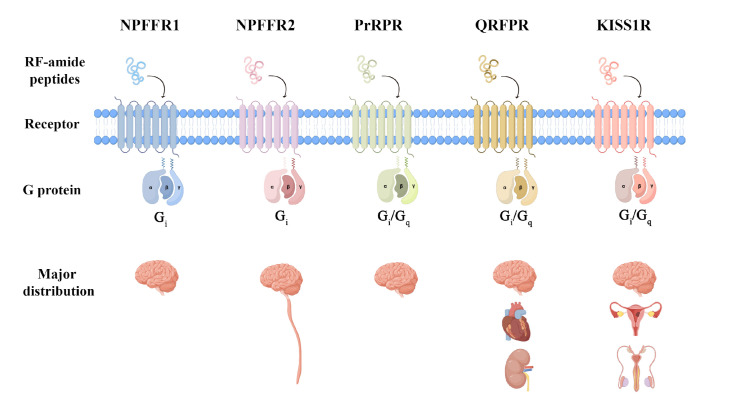
Overview of signaling pathways and distribution of the RF-amide receptor family. NPFFR1 is widely expressed in the central nervous system, and its coupled G protein is G_i_ protein. NPFFR2 is selectively distributed in various brain regions and the spinal cord, and its coupled G protein is G_i_ protein. PrRPR is expressed mainly in the brain, and its coupled G protein is G_i_ or G_q_ protein. QRFPR is expressed in the central nervous system and peripheral organs such as the kidneys and heart, and its coupled G protein is G_i_ or G_q_ protein. KISS1R is expressed in the central nervous system and reproductive system, and its coupled G protein is G_i_ or G_q_ protein. Created with Figdraw (ID: YIWUYefbf9). KISS1R: Kisspeptin receptor; NPFFR2: NPFF receptor 2; NPFFR1: NPFF receptor 1; PrRPR: prolactin-releasing peptide receptor; QRFPR: pyroglutamylated arginine–phenylalanine–amide peptide receptor; RF-amide: arginine–phenylalanine–amide.

The two neuropeptide FF receptor subtypes, NPFFR1 (GPR147) and NPFFR2 (GPR74), are receptors for endogenous NPFF, neuropeptide AF (NPAF), GnIH, and its homologs neuropeptide SF (NPSF, RFRP-1) and neuropeptide VF (NPVF, RFRP-3) (Bonini et al., 2000; Ubuka et al., 2013; **Additional Table 1**). The chromosomal localization and the distribution of human NPFFRs (hNPFFRs) were comprehensively described in previous work (Bonini et al., 2000; Goncharuk et al., 2004; Goncharuk and Jhamandas, 2008; Nguyen et al., 2020). Radiation hybrid mapping was performed to determine the chromosomal localization of the *hNPFFR1/2* genes. The *hNPFFR1* gene was shown to be situated on the long arm (q) of chromosome 10 at region 21, while the *hNPFFR2* gene is found on the long arm (q) of chromosome 4 between regions 13.2 and 13.3 (Bonini et al., 2000). In humans, the NPFFRs are predominantly expressed in the central nervous system (CNS) (**Figure 1**). The regions exhibiting the highest hNPFFR1 expression levels are the amygdala, hippocampus, and spinal cord, as determined by quantitative RT-PCR analysis of *hNPFFR1* mRNA in 24 human tissue samples (Nguyen et al., 2020). Immunohistochemistry of the human hypothalamus and surrounding areas revealed that hNPFFR1 is highly expressed in the posterior division of the bed nucleus of the stria terminalis and the zona incerta (Goncharuk et al., 2004). Meanwhile, high levels of hNPFFR2 were detected in the anterior amygdaloid area of the forebrain, the dorsomedial hypothalamic nucleus, and the dorsal motor nucleus of the vagus located in the medulla through quantitative RT-PCR and immunohistochemistry (Goncharuk and Jhamandas, 2008). However, the overall abundance of hNPFFR2 in the CNS system is considerably lower than that of hNPFFR1. Although the placenta shows the highest abundance of hNPFFR2 (**Additional Table 1**), hNPFFRs exhibit limited expression in other peripheral organs, with very low levels of hNPFFR mRNA being detected in the spleen, lungs, and kidneys (Nguyen et al., 2020).

**Additional Table 1:** Summaries of ligands, signaling pathways, distribution, and *in vivo* activity of RF-amide receptor family

	NPFFR1/2	PrRPR	QRFPR	KISS1R
Ligands	Endogenous ligands: NPFF (Bonini et al., 2000; Nguyen et al., 2020), NPAF (Bonini et al., 2000; Nguyen et al., 2020), GnIH (Ubuka et al., 2013), NPSF (Nguyen et al., 2020), NPVF (Nguyen et al., 2020) Representative exogenous ligands: 1DMe (Gicquel et al., 1992; Wei et al., 2001; Strnadová et al., 2024), RF9 (Simonin et al., 2006), dNPA (Yu et al., 2016), daY8Ra (Gouardères et al., 1996), BN-9 (Li et al., 2016), EN-9 (Wang et al., 2016), KGFF09 (Drieu la Rochelle et al., 2018)	Endogenous ligands: PrRP-20 (Kimura et al., 2000; Engström et al., 2003), PrRP-31 (Kimura et al., 2000; Engström et al., 2003) Representative exogenous ligands: lipidated PrRP analogs (Maletínská et al., 2015; Pražienková et al., 2017)	Endogenous ligands: 26RFa (Fukusumi et al., 2003; Jiang et al., 2003; Ukena et al., 2010), 43RFa (Fukusumi et al., 2003; Jiang et al., 2003; Ukena et al., 2010) Representative exogenous ligands: None	Endogenous ligands: KP-54 (Lee et al., 1996; Kotani et al., 2001a; Ohtaki et al., 2001; Aparicio, 2005), KP-14 (Lee et al., 1996; Kotani et al., 2001a; Ohtaki et al., 2001; Aparicio, 2005), KP-13 (Lee et al., 1996; Kotani et al., 2001a; Ohtaki et al., 2001; Aparicio, 2005), KP-10 (Lee et al., 1996; Kotani et al., 2001a; Ohtaki et al., 2001; Aparicio, 2005) Representative exogenous ligands: TAK-683 (Asami et al., 2013), TAK-448 (Matsui et al., 2014)
*In vivo* distribution	CNS (Goncharuk et al., 2004; Goncharuk and Jhamandas, 2008; Nguyen et al., 2020), placenta (Nguyen et al., 2020)	Brain (Roland et al., 1999; Ibata et al., 2000), adrenal medulla (Nieminen et al., 2003)	CNS (Prévost et al., 2019; Jiang et al., 2003), trigeminal ganglion (Jiang et al., 2003)	CNS (Lee et al., 1999; Muir et al., 2001; Mei et al., 2013; Trevisan et al., 2018), pituitary gland (Muir et al., 2001), reproductive organs (Muir et al., 2001; Mei et al., 2013; Trevisan et al., 2018)
Downstream signaling pathways	Primary pathways: G_i/o_ (Nourbakhsh et al., 2018)	Primary pathways: G_q/11_ (Kimura et al., 2000; Engström et al., 2003) Secondary pathways: G_i/o_ (Kimura et al., 2000; Engström et al., 2003)	Primary pathways: G_i/o_ (Fukusumi et al., 2003), G_q/11_ (Jiang et al., 2003)	Primary pathways: G_q/11_ (Kotani et al., 2001a; Zhang et al., 2013; Kim et al., 2020) Secondary pathways: G_i/o_ (Wu et al., 2024)
*In vivo* activities	Pain modulation (Yang et al., 2008; Zhang et al., 2018; Xu et al., 2020), neuroprotection (Dai et al., 2015; Yu et al., 2016), diet regulation (Hunt et al., 2011; Waqas et al., 2017; Lin and Chen, 2019; Zhang et al., 2022a), cancer development (Shin et al., 2022; Wu et al., 2023)	Stress management (Palkovits et al.,1980; Lin et al., 2002; Seal et al., 2002; Zhu and Onaka, 2003; Kovács et al., 2024), diet regulation (Lawrence et al., 2000, 2002; Pražienková et al., 2021; Talbot et al., 2023; Morgan et al., 2024), sleep modulation (Roky et al., 1995; Roland et al., 1999; Zhang et al., 2000, 2001; Steriade, 2005; Timofeev and Chauvette, 2011; Vas et al., 2023), pain modulation (Kalliomäki et al., 2004)	Feeding modulation (Granata et al., 2014; Prévost et al., 2015; El Mehdi et al., 2022; Le Solliec et al., 2022; Devère et al., 2025), cardiac control (Fang et al., 2009), pain modulation (Georgsson et al., 2014), reproduction (Navarro et al., 2006)	Reproduction (Chan et al., 2009; Ikegami et al., 2020; Khan and Chaudhry, 2021; Golyszny et al., 2022; Moenter and Evans, 2022; Wickramasuriya et al., 2022), diet regulation (Hudson and Kauffman, 2022), metabolism (Song et al., 2014), cancer development (Zhang et al., 2022b)

GnIH: Gonadotropin-inhibitory hormone; KISS1R: Kisspeptin receptor; NPAF: neuropeptide AF; NPFF: neuropeptide FF; NPFFR: NPFF receptor; NPSF: neuropeptide SF; NPVF: neuropeptide VF; PrRPR: prolactin-releasing peptide receptor; QRFPR: pyroglutamylated arginine-phenylalanine-amide peptide receptor.

The differential expression patterns of hNPFFRs in the CNS and peripheral organs provide valuable insights into their diverse physiological and pathological roles. Specifically, NPFFR1 is predominantly expressed in the human brain and spinal cord, suggestive of a role in pain regulation and neuroregulatory activities. Meanwhile, NPFFR2 is primarily expressed in the spinal cord, which suggests that it is involved in the transmission and regulation of pain signals (Nourbakhsh et al., 2018). This, in addition to the observation that the overall abundance of hNPFFR2 in the CNS is lower than that of hNPFFR1, implies that hNPFFR2 might have more specialized or peripheral roles, as indicated by its high expression in the placenta (Nguyen et al., 2020). Further research is needed to explore how this differential expression impacts their functional significance in the CNS and beyond.

#### NPFF receptors coupled G proteins

Both NPFFR1 and NPFFR2 commonly couple to G_i_/G_q_ proteins, resulting in the inhibition of adenylyl cyclase activity (Nourbakhsh et al., 2018; **Figure 1**). In NPFFR-expressing CHO cells, exposure to NPFF analogs led to a reduction in cyclic AMP levels (Kotani et al., 2001b), consistent with findings in NPFFR-expressing HEK293 cells (Elshourbagy et al., 2000) and SH-SY5Y neuroblastoma cells (Mollereau et al., 2005). Some scholars explored the specific G protein subunits involved in NPFFR signaling. Using a [^35^S]GTPγS binding assay, Gouardères et al. (2007) examined the effects of inhibitory peptides targeting Gα_i2/3_, Gα_o_, and Gα_s_ on hNPFFRs expressed in CHO cells. They found that agonist-stimulated [^35^S]GTPγS binding of NPFFR2 was inhibited by all three peptides, whereas NPFFR1 activity was only suppressed by the Gα_i3_ and Gα_s_ inhibitory peptides. These findings indicate that NPFFR1 can couple to Gα_i3_ and Gα_s_ proteins, while NPFFR2 is capable of coupling to Gα_i2/3_, Gα_o_, and Gα_s_ proteins.

#### The roles of the NPFF receptor signaling system in specific physiological processes

The NPFF signaling system has been implicated in a variety of physiological and pathological processes (**Additional Table 1**), including the regulation of pain (Yang et al., 2008; Zhang et al., 2018; Xu et al., 2020), nerve growth and regeneration (Dai et al., 2015; Yu et al., 2016), feeding behavior (Hunt et al., 2011; Waqas et al., 2017; Lin and Chen, 2019; Zhang et al., 2022a), and even the promotion of cancer development (Shin et al., 2022; Wu et al., 2023).

*Pain modulation*

The best-characterized role of this system is its opioid modulation ability in pain perception (Yang et al., 2008; Zhang et al., 2018; Xu et al., 2020). Pharmacological research has shown that NPFF-related peptides either enhance or counteract opioid-induced analgesic activity, depending on the site of administration. Specifically, they can produce opioid-like analgesia or even enhance opioid-induced analgesic effects when administered intrathecally, but reverse these effects when dispensed intracerebroventricularly (Yang et al., 2008). Based on such correlations, NPFF peptides can be modified to exert analgesic effects through interactions with both opioid and NPFF receptors (Zhang et al., 2018; Xu et al., 2020). DN-9, a compound combining opioid and NPFF pharmacophores, serving as both an opioid receptor agonist and an NPFF analog, exhibited potent analgesic effects when administered via spinal injection. RF-9, a non-selective NPFFR antagonist, enhanced the spinal analgesic effect of DN-9, indicating that DN-9 induces analgesia through opioid receptor activation while simultaneously modulating opioid effects via NPFFR activation (Zhang et al., 2018). Additionally, DN-9 inhibited gastrointestinal (GI) motility following intraventricular and intraperitoneal administration, an effect attributed to the simultaneous activation of NPFF and opioid receptors. RF-9 intensified the GI motility inhibition induced by DN-9, further supporting the involvement of NPFF receptors in modulating opioid receptor-mediated effects.

*Neural growth and regeneration*

NPFFR2 endogenously expressed in Neuro 2A cells can be activated by NPFF and dNPA, an NPFFR2 agonist, to stimulate neurite outgrowth through the G_i/o_ signaling pathway. Such neuritic processes rely on dNPA-induced phosphorylation of extracellular signal-regulated kinase (ERK) in Neuro 2A cells (Yu et al., 2016). When administered via subconjunctival injection, NPFF can promote the regeneration of the corneal nerve and enhance the healing of the corneal epithelium in diabetic mice. Additionally, NPFF can upregulate the expression of silent information regulator protein 1 and peroxisome proliferator-activated receptor gamma, thereby ameliorating the metabolic state of diabetic trigeminal neurons. Further RF-9 inhibition experiments demonstrated that the effects of NPFF in these processes were mediated via NPFFR2 and involved the activation of the ERK1/2 pathway (Dai et al., 2015).

*Regulation of food intake and energy metabolism*

The hypothalamic appetite control center regulates food consumption and energy homeostasis by integrating neural, hormonal, metabolic, and other signals from both internal and external sources, with NPFF/NPFFR2 signaling playing a crucial role in this process (Lin and Chen, 2019). A genetic association study reported that polymorphisms in the NPFFR2 locus are significantly associated with obesity predisposition (Hunt et al., 2011). In patients with obesity and mice fed a high-fat diet (HFD), the administration of NPFF can promote the activation of M2 adipose tissue macrophages, which show upregulated NPFFR2 expression, thus reducing inflammation and improving adipose tissue (AT) glucose tolerance. In HFD-fed mice, meanwhile, chronic treatment with NPFF decreases blood glucose levels while concurrently increasing insulin sensitivity (Waqas et al., 2017). NPFF/NPFFR2 signaling also participates in dietary heat production. The absence of the NPFF signal increases overall energy consumption and brown adipose tissue thermogenesis, a process regulated by neuropeptide Y neurons in the hypothalamic arcuate nucleus via NPFFR2 (Zhang et al., 2022a).

*Cancer development*

The NPFF system has also been implicated in cancer development. NPFFR2 was initially found to be highly expressed in hepatocellular carcinoma and promote tumor progression in a manner that was dependent on the RhoA/ROCK pathway, which plays an important role in cytoskeleton remodeling and cell movement. Specifically, NPFFR2 positively regulates the ROCK/F-actin/YAP signaling axis, leading to YAP signaling activation, and, consequently, hepatocellular carcinoma progression (Shin et al., 2022). Moreover, high expression of NPFFR2 was associated with low survival rates in patients with epithelial ovarian cancer (EOC). In SK-OV-3 cells (a human EOC cell line), NPFF treatment activated ERK1/2 signaling via NPFFR2, leading to the upregulation of the expression of matrix metalloproteinase-9, a protein with a known role in tumor invasion and metastasis. These findings strongly support that this signaling pathway promotes EOC cell invasion (Wu et al., 2023).

### Prolactin-releasing peptide receptor

#### Ligands, chromosomal localization, and distribution of the prolactin-releasing peptide receptor

Prolactin-releasing peptide receptor (PrRPR, GPR10) is targeted by two isoforms of PrRP of differing length but equal activity, namely, prolactin-releasing peptide-31 (PrRP-31) and PrRP-20 (Hinuma et al., 1998; **Additional Table 1**). Both isoforms are generated through the cleavage of the PrRP precursor. In mammals, PrPPR is encoded by the *PRLHR* gene. In humans, *PRLHR* is located on the long arm (q) of chromosome 10 spanning regions 25.3 to 26.1 (Marchese et al., 1995). Radio labeling, *in situ* hybridization, and immunohistochemistry have shown that PrRPR is highly expressed in hypothalamic nuclei (paraventricular nucleus [PVN], periventricular hypothalamic nucleus [PEVN], dorsomedial hypothalamic nucleus [DMN]), the medulla oblongata (nucleus of the solitary tract [NTS] and area postrema [AP]), and the thalamus (reticular nucleus of the thalamus [RT]), while an intermediate level of expression is observed in the ventromedial hypothalamic nucleus and the anterior pituitary (Roland et al., 1999; Ibata et al., 2000). *PrRPR* mRNA is also detected at very low levels in peripheral tissues, such as the adrenal medulla of rats (Roland et al., 1999; Ibata et al., 2000; Nieminen et al., 2003).

#### Prolactin-releasing peptide receptor-coupled G proteins

Studies have confirmed that PrRPR can couple with G_i/o_ or G_q/11_ proteins and activate the respective downstream signaling pathways (Kimura et al., 2000; Engström et al., 2003; **Figure 1**). Engstrom et al. (2003) used the [^35^S]GTPγS binding assay to evaluate the coupling selectivity of PrRPR to G_i/o_ proteins. They observed increased [^35^S]GTPγS binding when PrRPR was stimulated with PrRP (the binding of PrRP-20 or PrRP-31), indicative of G_i/o_ protein activation. This was further confirmed via the application of pertussis toxin, a G_i/o_ protein inhibitor, which significantly suppressed PrRP-induced signal transduction by more than 80% (Engström et al., 2003). Kimura et al. (2000) found that the PrRP-induced activation of c-Jun N-terminal kinase (JNK) was dependent on protein kinase C, suggesting that G_q/11_ proteins play a role in this process by mediating protein kinase C activation.

Moreover, Li et al. (2024) elucidated the structural basis underlying PrRPR-G_q_ and PrRPR-G_i_ interactions, finding that the coupling of PrRPR with G_q_ and G_i_ proteins exhibits distinct conformational differences. In the PrRPR-G_q_ complex, transmembrane domain 6 (TM6) undergoes significant outward displacement to promote G_q_ binding, while in the PrRPR-G_i_ complex, TM6 adjusts inward to accommodate the G_i_ protein. In addition, the “wavy hook” region in the PrRPR-G_q_ complex exhibits more extensive polar interactions than that in the PrRPR-G_i_ complex, supporting selective G_q_ coupling. Overall, these conformational adjustments explain how PrRPR achieves selective G protein coupling, undergoing distinct structural adaptations that facilitate the activation of targeted signaling pathways (Li et al., 2024).

#### The roles of the prolactin-releasing peptide signaling system in specific physiological processes

The PrRP system was named for its ability to stimulate prolactin secretion by lactotropic cells (Hinuma et al., 1998). However, it has since been shown that it also participates in several other physiological activities (**Additional Table 1**), especially in the restriction of calorie intake (Lawrence et al., 2000, 2002; Chandel, 2021; Pražienková et al., 2021; Talbot et al., 2023; Morgan et al., 2024), stress management (Palkovits et al., 1980; Lin et al., 2002; Seal et al., 2002; Morales and Sawchenko, 2003; Zhu and Onaka, 2003; Kawakami et al., 2008; Onaka et al., 2010; Maniscalco and Rinaman, 2017; Davis and Grill, 2018; Holt and Rinaman, 2022; Kovács et al., 2024), and sleep regulation (Roky et al., 1995; Roland et al., 1999; Zhang et al., 2000, 2001; Steriade, 2005; Timofeev and Chauvette, 2011; Vas et al., 2023).

*Suppression of appetite and restriction of energy expenditure*

Most relevant studies to date have indicated that PrRP-PrRPR/GPR10 signaling exerts significant anorexigenic effects, meaning that it plays a key role in suppressing appetite and reducing energy expenditure (Lawrence et al., 2000, 2002; Pražienková et al., 2021; Talbot et al., 2023; Morgan et al., 2024). Intracerebroventricular (ICV) PrRP administration in rats and mice resulted in the suppression of appetite, leading to weight loss (Lawrence et al., 2000). However, this anorexigenic effect did not disrupt normal feeding behavior, suggesting that the natural eating-to-satiety remained unaffected (Lawrence et al., 2000, 2002). Correspondingly, PrRPR-knockout (KO) mice displayed obesity-related symptoms (Pražienková et al., 2021). In addition, a recent study involving GPR10 and NPFFR2 double-KO mice showed that the absence of PrRPR and NPFFR2 led to negative manifestations, including impaired glucose tolerance, excessive insulin secretion, and suboptimal insulin action. These effects exhibited gender-specific variations, which were most prominent under HFD conditions (Morgan et al., 2024). In a recent study on rare, obesity-related PrRPR variants, researchers generated a mutation at the p193 site of PrRPR, a highly conserved residue located at the extracellular end of TM4. They found that this mutation affected the function of the active pocket of the receptor, preventing ligand (PrRP-31) binding and impairing G protein-dependent signal transduction, eventually resulting in obesity (Talbot et al., 2023).

PrRPR also plays an important role in lipid metabolism, which is a crucial component in the maintenance of energy homeostasis (Pražienková et al., 2021). Energy homeostasis depends both on energy expenditure as well as the ability to regulate energy intake through metabolic pathways, including lipid metabolism (Chandel, 2021). In a recent study on GPR10 KO mice of different genders, male mice showed significant adipose tissue accumulation under a standard diet, while under a HFD, male mice exhibited downregulation of adipogenic gene expression. In female mice, there was no apparent body weight gain relative to their wild-type (WT) counterparts under either diet nor were there any noticeable fluctuations in free fatty acid levels. The former may indicate that a compensatory increase in lipid metabolism-related genes counteracted fat accumulation, while the latter may suggest that insulin function was impaired, which suppressed lipolysis (Pražienkováet al., 2021). The above perspectives emphasize that although the effects vary by gender, the PrRP/PrRPR system plays an undeniable role in the overall regulation of lipid metabolism.

*Stress management*

PrRP and PrRPR are co-expressed in several stress-related brain regions, such as the hypothalamus and the hindbrain (Davis and Grill, 2018). External acute stress can activate neurons where PrRP and PrRPR work in conjunction (Morales and Sawchenko, 2003). Key areas involved in stress regulation, such as the PVN and bed nucleus of the stria terminalis, were found to be enriched with PrRP neurons (Maniscalco and Rinaman, 2017; Holt and Rinaman, 2022). Although double in situ hybridization showed that most PrRP-expressing neurons in the PVN do not co-express corticotropin-releasing hormone (CRH), the ICV administration of PrRP-activated CRH neurons in the PVN (as indicated by c-Fos expression), triggering the release of stress-related hormones such as adrenocorticotropic hormone and corticosterone (Lin et al., 2002; Seal et al., 2002; Kovács et al., 2024). Moreover, PrRP-expressing neurons in the bed nucleus of the stria terminalis, which co-express CRH, directly regulated the hypothalamic–pituitary–adrenal axis through the PVN (Palkovits et al., 1980; Lin et al., 2002). These findings emphasized that PrRP is involved in the modulation of CRH neurons and also influences the hypothalamic–pituitary–adrenal axis in stress regulation (Zhu and Onaka, 2003). Interestingly, the PrRPR-mediated regulation of stress responses may have a complex interaction with the previously mentioned regulation of food intake and energy expenditure, with the two regulatory mechanisms potentially influencing each other (Kawakami et al., 2008). For example, PrRP KO mice under stress exhibit diminished oxygen expenditure (Onaka et al., 2010). Such interactions can be further explored through experiments using *GPR10* or *PrRP* gene-edited mice.

*Control of circadian rhythmicity and sleep mode*

The distribution of PrRPR in multiple sleep-related brain regions indicates that the PrRP signaling system participates in circadian rhythmicity and sleep mode control. The thalamic reticular nucleus (Rt), composed of GABAergic neurons, displays high levels of PrRPR expression (Roland et al., 1999). The Rt region filters ascending and descending neural signals before they reach the cerebral cortex, thereby contributing to the regulation of the transition from wakefulness to sleep (Steriade, 2005; Timofeev and Chauvette, 2011). Early studies investigating the effect of PrRP on sleep patterns in rats through ICV injection found that PrRP exerts a dose-dependent effect on sleep and prolactin (PRL) secretion. Specifically, rapid eye movement (REM) sleep and plasma PRL levels were promoted at low PrRP doses, while non-REM (NREM) sleep was preferentially induced at high doses (Zhang et al., 2000, 2001). These findings also suggested that PrRP is closely related to PRL secretion. PRL participates in the regulation of REM, especially by modulating day–night rhythms via its effects on the hypothalamus (Roky et al., 1995; Zhang et al., 2000, 2001). Recent investigations of the association between stress and sleep disorders involving PrRP reported that PrRP, as a stress regulator, influences sleep through complex neural circuits. Sleep deprivation led to an increase in PrRP expression in the dorsal medial hypothalamic nucleus. Sustained stress can result in the excessive activation of PrRP, while a reduction in GPR10 expression may lead to the dysregulation of the PrRP signaling pathway-mediated stress response, eventually leading to sleep disturbances (Vas et al., 2023).

### Pyroglutamylated arginine–phenylalanine–amide peptide receptor

#### Ligands, chromosomal localization, and distribution of the pyroglutamylated arginine–phenylalanine–amide peptide receptor

The QRFP receptor (QRFPR, GPR103) is targeted by the 26-amino acid RF-amide peptide (26RFa) and its N-terminally extended form, the 43-amino acid RF-amide peptide (43RFa, QRFP) (**Additional Table 1**). Both peptides are generated through the processing of a common precursor, pro-QRFP (P518), and the amino acid sequence of 26RFa exhibits a high degree of conservation in mammals (Ukena et al., 2010). Activation experiments using partial 26RFa C-terminal residues demonstrated that the integrity of the full sequence (or the longer 43RFa) is essential for achieving potent binding to QRFPR (Fukusumi et al., 2003; Jiang et al., 2003). The *QRFPR* gene is located at the long arm (q) of chromosome 4 (121,328,642–121,381,059 bp) (data acquired from Ensembl). The expression of human QRFPR was mapped in detail in human tissue. The highest QRFPR mRNA expression detected in the brain was accompanied by the coordinated expression of its ligand precursor (pro-QRFP, P518) in the same brain regions (**Figure 1**). Abundant QRFPR expression was detected in the hypothalamus, trigeminal ganglion (TG), and vestibular nucleus (VN) (Jiang et al., 2003). Regarding peripheral expression, QRFPR is predominantly expressed in fetal bones, testes, kidneys, and heart (Jiang et al., 2003; **Figure 1**). Prévost et al. investigated the distribution of the QRFPR system (26RFa-QRFPR) in the human digestive system and found that QRFPR and its ligand (26RFa) are distributed throughout the entire intestine, with the highest levels found in the stomach and duodenum. This system is also expressed in pancreatic islets, primarily in pancreatic β-cells (Prévost et al., 2019).

#### Pyroglutamylated arginine–phenylalanine–amide peptide receptor-coupled G proteins

QRFPR has been confirmed to couple to G_q_ or G_i/o_ proteins, leading to the activation of distinct downstream signaling pathways (Fukusumi et al., 2003; Jiang et al., 2003; Ma et al., 2021; **Figure 1**). Jiang et al. (2003) reported that QRFPR naturally couples with G_q_ proteins, and its activation by P518, its ligand precursor, is not significantly affected by other G protein subtypes. In a separate study, the stimulation of GPR103-expressing CHO cells with QRFP resulted in a dose-dependent restriction of cAMP production, with the full-length QRFP (43RFa) exerting the most potent inhibitory effect. These results suggested that QRFPR couples to G_i/o_ proteins, a conclusion supported by the observed dose-dependent inhibitory effect of 43RFa (Fukusumi et al., 2003). A recent study also revealed the G protein-coupling preferences of QRFPR, where it was observed that QRFPR simultaneously activated signaling pathways associated with Gα_q_ and Gα_i/o_ proteins upon binding to 26RFa, leading to intracellular Ca^2+^ responses mediated by Gα_q_ and Gβγ, as well as Gα_i/o_-mediated suppression of cAMP production (Ma et al., 2021).

The structural details of QRFPR-Gq interactions have been resolved in two independent studies (Iwama et al., 2024; Jin et al., 2024). Jin et al. (2024) provided a detailed description of how QRFPR interacts with the G_q_ protein. They demonstrated that QRFPR primarily associates with the “wavy hook” region of the α5 helix on the Gα_q_ subunit through its cytoplasmic cavity, while also engaging the hydrophobic groove on Gα_q_ with its ICL2 region. The extended portions of TM5 and TM6 in QRFPR further reinforce binding stability with G_q_ through additional salt bridges (Jin et al., 2024).

#### The roles of the pyroglutamylated arginine–phenylalanine–amide peptide receptor signaling system in specific physiological processes

The QRFPR system (QRFP and GPR103) is extensively expressed in the hypothalamus (Jiang et al., 2003), a brain region closely associated with the regulation of diet-related behaviors (Roger et al., 2022). This indicates that the QRFP-QRFPR system may be a key neural mechanism in food intake regulation and blood sugar (glucose) balance (**Additional Table 1**). These physiological effects render QRFPR a potential target for the treatment of eating disorder-related diseases such as obesity and diabetes.

*Regulation of appetite and glucose homeostasis*

In contrast to the previously described effects mediated by the PrRP system, the impact of 26RFa/43RFa mediated by QRFPR demonstrates significant and persistent orexigenic (appetite-stimulating) activity (Cook et al., 2022). The oxygenic effect of QRFP prompts the intake of more food and the conversion of the ingested food (especially carbohydrates) into glucose, which subsequently enters the bloodstream and leads to an increase in postprandial blood glucose levels (Devère et al., 2025). QRFP in the gut can partially stimulate β-cells in the pancreas to secrete insulin to alleviate the glucose load in the blood (Prevost et al., 2015). These roles are supported by extensive data accumulated over the past 10 years.

A study published in 2014 showed that QRFPR, through its ligands 43RFa and 26RFa, not only promotes appetite but also participates in the regulation of insulin secretion and the protection of β-cell function. Both 43RFa and 26RFa were found to have positive effects on the survival and apoptosis of pancreatic β-cells and human pancreatic islets, 43RFa by promoting insulin secretion and increasing glucose uptake, and 26RFa by inhibiting insulin secretion through the Gα_i/o_ pathway (Granata et al., 2014). Prévost et al. (2019), in addition to investigating the distribution of the QRFPR system in the human digestive system, as previously mentioned, also compared the response of the system between patients with obesity and healthy controls during an oral glucose tolerance test. The difference in plasma 26RFa levels observed between the two groups indicated that the system may become ineffective in controlling glucose homeostasis in obese patients, potentially leading to increased 26RFa levels as a compensatory mechanism. Additionally, a positive correlation was found between plasma 26RFa levels and the body mass index, insulin secretion, and insulin resistance, suggesting that the QRFPR system may be tightly associated with the decreased insulin sensitivity and increased insulin resistance commonly seen in obesity and type 2 diabetes mellitus (Prévost et al., 2019). The mechanism underlying the effect of this system on central glucose modulation involves the transmission of insulin signals to the brain, thereby influencing glucose homeostasis (El Mehdi et al., 2022). However, such modulatory effects seem to be short-term, given that rapid central administration of QRFP can lead to a temporary decline in blood glucose via a concomitant increase in insulin secretion, while prolonged use cannot achieve long-term control of blood sugar in mice with obesity or diabetes (Le Solliec et al., 2022). These findings imply that QRFP has potential as a short-term treatment for hyperglycemia. Future research should delve deeper into the specific mechanisms of action of this system in acute metabolic regulation and explore strategies for achieving sustainable therapeutic outcomes.

### Kisspeptin receptor

#### Ligands, chromosomal localization, and distribution of the kisspeptin receptor

The Kisspeptin receptor (KISS1R, GPR54) is targeted by peptides encoded by the human KISS1 gene. This gene generates a 145-amino acid precursor, prepro-kisspeptin, which undergoes a series of cleavages, yielding kisspeptin fragments of differing lengths. The primary product of this cleavage is the 54-amino acid fragment, kisspeptin-54 (KP-54), initially named metastin for its inhibitory effect on cancer metastasis. Shorter fragments—KP-10, KP-13, and KP-14—are generated through further cleavage of KP-54 (Lee et al., 1996; Kotani et al., 2001b; Ohtaki et al., 2001; Aparicio, 2005; **Additional Table 1**). According to Ensembl, the KISS1R gene is located at the short arm (p) of chromosome 19, spanning 917,287 bp through 921,005 bp. Regarding distribution, KISSR1 and its ligands were initially determined to be extensively expressed in the placenta. In the CNS, KISS1R was found in multiple regions, particularly in the cerebral cortex (including pyramidal cells), amygdala, nucleus accumbens, hippocampus, cingulate gyrus, hypothalamus, cerebellum (including Purkinje cells), medulla oblongata, and basal ganglia, as determined using quantitative PCR assays (Lee et al., 1999; Muir et al., 2001; Mei et al., 2013; Trevisan et al., 2018). KISS1R was also abundantly present in the pituitary gland, which is regulated by the hypothalamus. In the peripheral system, high-level expression of KISS1R was found in the reproductive organs (testes and ovaries), reflecting its role in regulating reproductive function, hormone balance, and sexual development, whereas low-level expression was observed in lymphocytes, pancreas, and adipose tissue (Muir et al., 2001; Mei et al., 2013; Trevisan et al., 2018).

#### Kisspeptin receptor-coupled G protein

KISS1R is a recognized G_q/11_-coupled receptor that activates phospholipase C (PLC), triggering the generation of IP3 and DAG. This leads to an increase in the level of intracellular Ca^2+^ and the activation of calcium-dependent signaling pathways, thereby influencing a variety of cellular responses (Kotani et al., 2001a; Zhang et al., 2013; Kim et al., 2020; **Figure 1**). Our group has previously elucidated the details underlying the KISS1R-G_q/11_ molecular interaction (Wu et al., 2024). In our work, we measured Gq protein coupling in KISS1R signaling pathways. Specifically, we measured the response of WT KISS1R and various KISS1R mutants to stimulation by KP-54 through IP1 accumulation assays, a method that can characterize the strength of receptor signaling. The results showed that mutations in different transmembrane domains and extracellular loops of KISS1R can impact the ability of the receptor to mediate KP54-induced IP1 accumulation. Specific mutations in critical regions, such as R297A in extracellular loop 3, severely impair receptor function, while others only partially reduce signaling (Wu et al., 2024).

Despite the understanding of the KISS1R-Gi signaling pathway being limited, our research demonstrated that KISS1R can also activate the G_i_ protein (**Figure 1**). In this instance, we used TAK-448, a kisspeptin analog, as the ligand to evaluate the impact of mutations in the KISS1R receptor through a cAMP inhibition assay. We found that the WT KISS1R strongly inhibits cAMP production, which is a hallmark of G_i_ protein coupling (Wu et al., 2024). This indicates that when activated by TAK-448, KISS1R inhibits cAMP production by engaging with the G_i_ pathway. The effects of mutations such as R297A, which significantly mitigate cAMP inhibition, suggest that these specific residues are critical for G_i_ protein coupling. Given that wild-type KISS1R suppresses cAMP generation, whereas certain mutations disrupt this function, directly points to Gi involvement.

#### The roles of the Kisspeptin receptor signaling system in specific physiological processes

Kisspeptin was initially discovered as a product of the metastasis suppressor gene *KISS1*, which suggested that the KP-KISS1R system is associated with the inhibition of tumor metastasis (Kotani et al., 2001a; Ohtaki et al., 2001; Nash and Welch, 2006). Subsequently, this system was found to exert a variety of physiological functions (Song et al., 2014; Navarro, 2020; Hudson and Kauffman, 2022; Guzman et al., 2022; Zhang et al., 2022b; **Additional Table 1**), particularly the regulation of reproductive function, including promoting sexual maturity and ensuring fertility. Below, we highlight its contributions to sexual maturation and reproductive capacity.

*Regulation of pubertal initiation and progression*

KISS1R plays an essential role in the hypothalamic–pituitary–gonadal axis, regulating the secretion of gonadotropin-releasing hormone (GnRH) through its ligand kisspeptin (Khan and Chaudhry, 2021; Gołyszny et al., 2022). Studies have shown that both humans and mice carrying *KISS1R* or *KISS1* gene mutations exhibit varying degrees of reproductive dysfunctions, which were attributed to impaired or complete loss of GnRH secretion (Chan et al., 2009; Ikegami et al., 2020). GnRH is essential for regulating gonadal development and sex hormone production. Its continued secretion, beginning during embryonic development and continuing through adulthood, is fundamental for ensuring proper reproductive function (Wickramasuriya et al., 2022).

Multiple mammalian experiments have demonstrated that the KP-KISS1R system promotes the depolarization, and, consequently, the activation of GnRH neurons in the hypothalamus through the G_q_ pathway. This triggers the pulsatile release of GnRH, thereby initiating puberty. Moreover, *KISS1R* and *KISS1* mRNA levels in the hypothalamus significantly increase as puberty progresses (Navarro et al., 2004; Han et al., 2005; Shahab et al., 2005; Sun et al., 2007; Keen et al., 2008). The initiation and progression of puberty signify the maturity of the reproductive system, during which GnRH controls the development of the gonads and the production of sex hormones by promoting the secretion of gonadotropins (luteinizing hormone and follicle-stimulating hormone) by the pituitary gland. Luteinizing hormone and follicle-stimulating hormone further act on the gonads (e.g., testes and ovaries), promoting the production of sex hormones such as testosterone and estrogen. These sex hormones trigger a series of physical changes during adolescence, including the appearance of secondary sexual characteristics such as breast development and testicular and penile enlargement, and ensure that individuals have reproductive ability (Keen et al., 2008; Dwyer and Quinton, 2019; Fuqua and Eugster, 2022; Moenter and Evans, 2022).

*Regulation of reproductive function*

The effects of the KP-KISS1R system on fertility vary between males and females due to differences in reproductive physiology. Normal *KISS1/KISS1R* gene expression is required for the maturation of male reproductive organs (Khan et al., 2022). Notably, specific mutations can result in underdeveloped external genitalia and undescended testes (Chan et al., 2009). A study on rat testicular tissue found a positive correlation between KISS1 expression and testosterone levels (Aytürk et al., 2017). In addition, KISS1R and its ligands were found in distinct regions within male sperm through immunodetection. Their distribution in different regions of sperm suggests that KP and KISSSR may regulate multiple sperm functions and activities, including flagellar movement for motility and energy and motion functions, thus contributing to sperm hyperactivation and facilitating fusion with the oocyte (Blasco et al., 2019). These functions are crucial for the normal workings of sperm and successful fertilization.

Because women have to both manage reproductive processes tied to the menstrual cycle and bear the responsibility of pregnancy and its progression, the role of the KISS1R system is substantially more intricate and multifaceted in the female reproductive system compared with that in males. The maintenance of the dominant follicle in the early menstrual cycle is vital for the maturation of the egg (Candelaria et al., 2020). In a study of egg donors for *in vitro* fertilization, KISS1R and its ligands were found to be differentially expressed between parietal and cumulus cells (García-Ortega et al., 2014). In turn, the incomplete expression of KISS1R was found to induce premature ovarian failure (POF) in mice (Gaytan et al., 2014). Combined, these observations highlight the importance of the KISS1R system in follicle maintenance and oocyte maturation. Follicle maturation results in a sharp secretion of LH, triggering ovulation when a certain threshold is achieved (Medeiros et al., 2021). This preovulatory LH surge can be induced by administering kisspeptin, either centrally or peripherally (Hrabovszky et al., 2010; Owen et al., 2021). This process is mediated through the activation of KISS1R-expressing GnRH neurons (Stevenson et al., 2022). The potent stimulatory effect of kisspeptin on gonadotropin secretion has been validated across multiple species (Shahab et al., 2005; Adams et al., 2018; Garcia et al., 2018; Ilahi and Haq, 2021; Anderson and Millar, 2022). During pregnancy, the levels of KP (mainly KP-54) in the mother’s blood greatly increase, primarily originating from the trophoblast cells of the placenta (Tsoutsouki et al., 2022). Conversely, low serum levels of KP can increase the risk of fetal loss, particularly in initial and late pregnancy periods (Sullivan-Pyke et al., 2018; Silva et al., 2023). These types of correlations imply that KP can serve as a candidate marker for identifying the risk of pregnancy loss.

## Drug Development for Arginine–Phenylalanine–Amide Receptors

The above-mentioned RF-amide receptor signaling systems have demonstrated therapeutic potential in several pathological conditions. This promise has attracted significant research efforts to modify and optimize their ligands for the development of targeted drugs.

### Structure-activity relationship studies for the development of arginine–phenylalanine–amide peptide analogs

To date, the development of most RF-amide analogs has been based on structure-activity relationship (SAR) studies (Gicquel et al., 1992, 1994; Gouardères et al., 1996, 1998; Wei et al., 2001; Simonin et al., 2006; Maletínská et al., 2013; Liu and Herbison, 2014; Bihel et al., 2015; Li et al., 2016; Wang et al., 2016; Elhabazi et al., 2017; Lin et al., 2017; Strnadová et al., 2024), which involves examining the relationship between a compound’s chemical structure and its biological activity. This approach provides a theoretical foundation for optimizing the potency, selectivity, and pharmacokinetic properties of molecules. In SAR analyses, the chemical groups of a ligand are systematically modified to observe how these changes affect target binding and biological activity, making it particularly useful in early drug development (Guha Rajarshi, 2013; Kenakin et al., 2019).

#### Structure-activity relationship studies for neuropeptide FF receptor ligands

Numerous SAR-based studies have investigated NPFFR ligands, including NPFF-based agonists and antagonists (Gicquel et al., 1992, 1994; Gouardères et al., 1996, 1998; Wei et al., 2001; Strnadová et al., 2024), small-molecule agonists and antagonists (Bihel et al., 2015; Elhabazi et al., 2017), and dual-target ligands (Li et al., 2016; Wang et al., 2016; Drieu la Rochelle et al., 2018). The early development of NPFF analogs involved enhancing their resistance to peptidases through structural modifications, such as replacing N-terminal amino acids with D-amino acids or adding methyl groups, ultimately yielding the stable agonist 1DMe and its analogs (Gicquel et al., 1992; Wei et al., 2001; Strnadová et al., 2024). However, evidence from SAR studies indicates that truncating N-terminal residues only leads to a moderate decrease in receptor affinity, while C-terminal residues are crucial for receptor binding (Gicquel et al., 1994). SAR studies have also guided the design of peptide antagonists such as daY8Ra (Gouardères et al., 1996) and dansyl-PQR-NH_2_ (Gouardères et al., 1998; Lin et al., 2017), which exhibit strong resistance to enzymatic digestion under physiological conditions and can effectively block the activity of NPFF receptors. Another example is the previously mentioned non-selective antagonist RF-9. Its development involved the structural modification of N-terminal capped dipeptides, and it was designed to have good receptor binding ability and blood–brain barrier permeating ability (Simonin et al., 2006). However, under certain conditions, it also exhibits agonist properties. For example, in fasted mice, RF-9 shows appetite-stimulating effects similar to [Tyr^1^] NPFF (Maletínská et al., 2013). In addition, RF-9 has been found to activate KISS1R, which, in turn, affects GnRH neuron activity (Liu and Herbison, 2014).

The development of small-molecule NPFFR agonists, such as quinazolino-guanidine and aminoguanidine derivatives, relies on SAR analysis to optimize their binding affinity and receptor selectivity. The aim of structural changes is to enhance the pharmacological properties of these molecules, such as reversing pain hypersensitivity without causing negative side effects. Regarding small molecule antagonists, modifying the chemical structure of the non-selective antagonist RF-9 led to the development of RF-313 (Elhabazi et al., 2017). This compound has shown potent NPFFR1 antagonistic activity in *in vitro* experiments. It can also enhance the analgesic effect of opioid drugs, thereby mitigating opioid-induced pain hypersensitivity. Simultaneously, RF-313 exhibits higher receptor selectivity compared to RF-9, showing reduced non-specific binding to other receptors in the same family, such as KISS1R (Bihel et al., 2015).

SAR-based studies have effectively promoted the development of bifunctional ligands such as EN-9 and BN-9. These ligands simultaneously act on opioid receptors (e.g., μ opioid receptor, δ opioid receptor, and κ opioid receptor) and NPFF receptors, providing effective analgesic effects and reducing drug resistance and side effects (Li et al., 2016; Wang et al., 2016). These ligands delay tolerance development in certain pain models and reduce the side effects of opioid drugs, such as constipation and respiratory depression. KGFF09, a μ-opioid receptor agonist and NPFFR antagonist can provide long-lasting analgesia without inducing tolerance or withdrawal reactions (Drieu la Rochelle et al., 2018).

***Structure-activity relationship studies for prolactin-releasing peptide receptor ligands***

In SAR studies concerning PrRP peptide analogs, researchers initially observed that truncating the N-terminus decreased the binding affinity of PrRP for PrRPR (Roland et al., 1999; Pražienková et al., 2019). A 13-peptide fragment of PrRP (19–31) is the shortest sequence that retains biological activity. It exhibits agonist properties while maintaining its affinity for the intact PrRPR (Boyle et al., 2005). In contrast, PrRP (25–31) is the minimal truncated form of the PrRP molecule, containing only the seven crucial amino acids at the C-terminus. While this fragment can bind to PrRPR, it cannot effectively activate the receptor, and its affinity is reduced by two orders of magnitude compared to the complete PrRP20 or PrRP31 (Roland et al., 1999; Pražienková et al., 2019). The C-terminus is vital for maintaining ligand binding activity, as replacement of the terminal amide group results in a complete loss of receptor affinity (Roland et al., 1999). Specifically, the arginine (Arg) and phenylalanine (Phe) residues located at the C-terminus are essential for sustaining binding affinity (D’Ursi et al., 2002). For instance, it was shown that substituting Arg26 or Arg30 significantly reduces binding affinity, whereas the incorporation of larger hydrophobic or aromatic side chains at the Phe31 position can preserve or even enhance both binding affinity and agonist activity (Roland et al., 1999). Some non-natural amino acids—such as dichlorophenylalanine (PheCl_2_^31^) and naphthylalanine (Nal)—have been shown to retain or increase receptor-binding potency while also preserving agonist functionality (Maletínská et al., 2011).

The addition of fatty acids of various lengths, such as those containing 8 to 16 carbon chains, to the N-terminus of PrRP enhances its stability and extends its half-life (Maletínskáet al., 2015). This modification holds great potential in drug development, as it increases the ability of PrRP to cross the blood–brain barrier (Brasnjevic et al., 2009), making it suitable for the development of drugs aimed at regulating appetite and metabolism. The lipidated PrRP analog, LiPR, retains a high binding affinity for PrRPR and exhibits a longer duration of action both *in vivo* and *in vitro* (Maletínská et al., 2015; Pražienková et al., 2017). Recently, LiPR harboring a N-γ-palmitoyl modification at Lys^10^ of the sequence has been shown to exert a protective effect in mice fed a HFD. This analog exhibits neuroprotective effects similar to the anti-obesity drug liraglutide, a glucagon-like peptide-1 analog, promoting the survival of hypothalamic adult neurons and increasing the number of adult neural stem cells (Jörgensen et al., 2024). In another LiPR development project focused on achieving significant anti-obesity effects, researchers modified PrRP-31 with an 18-carbon chain (C18), yielding dual-action agonists of PrRPR and NPFFR2. This modification significantly reduced the weight of obese mice by suppressing their appetite, an effect that persisted for up to a week after treatment (Alexopoulou et al., 2022).

#### Structure-activity relationship studies for pyroglutamylated arginine–phenylalanine–amide peptide receptor ligands

Two endogenous ligands of QRFPR, 26RFa and 43RFa, share the same C-terminal region, which contains residues crucial for receptor activity (do Rego et al., 2006; Leprince et al., 2017). Compared to 43RFa, 26RFa boasts a more streamlined structure, which facilitates its study and modification. The core structure of 26RFa retains key bioactivities, such as appetite stimulation and glucose homeostasis regulation, imparting it with considerable therapeutic potential and making it a valuable target for advanced biomedical research (do Rego et al., 2006; Chartrel et al., 2016).

Building on this, a fragment of 26RFa—26RFa(20–26)—composed of the last seven residues of its C-terminus was subjected to SAR analysis, with the results showing that the Phe^24^-Arg^25^-Phe^26^-NH_2_ triad within the heptapeptide was crucial for ligand bioactivity (Alim et al., 2018; Lefranc et al., 2021).

Given the importance of the Phe^24^ and Phe^26^ sites for receptor binding and the maintenance of ligand activity, these Phe residues are highly sensitive to structural modifications and typically intolerant to substitution (Chartrel et al., 2016; Lefranc et al., 2021). However, the receptor binding activity of these residues can be preserved or enhanced through precise point mutations and small-molecule chemical modifications. For example, a para chloro-phenylalanine [Phe (4-Cl)] substituent can be introduced into the benzene ring of Phe^24^ to enhance its receptor binding ability, while Phe^26^ can be modified with a similar para substitution using p-chlorophenylalanine (PCP26). This substitution reduces molecular spatial barriers, facilitating its entry into the binding pocket of QRFPR, thereby maintaining or enhancing ligand activity. Moreover, the EC_50_ for receptor activation can be significantly lowered by replacing Phe^22^ in 26RFa with tetrahydroisoquinoline-3-carboxylic acid (Tic), which is among the most effective structural modifications for enhancing the receptor activation potency of 26RFa analogs (Lefranc et al., 2021).

The Arg^25^ residue has been found to interact with the Gln^125^ residue of QRFPR, which is thought to be pivotal for the binding of 26RFa to and the activation of QRFPR. Alim et al. (2018) revealed that the introduction of a *ω-methyl* modification on Arg^25^ enhanced the modified peptide’s efficacy by 25-fold compared to the original heptapeptide. Furthermore, *in vivo* studies demonstrated that this modification produces a delayed hypoglycemic effect, akin to that of 26RFa.

#### Structure-activity relationship studies for Kisspeptin receptor ligands

KP-10 is the C-terminal decapeptide of full-length kisspeptin, which contains the core region needed for GPR54 binding and activation. Its simplified molecular structure makes it ideal for SAR studies, facilitating the exploration of key structural features associated with receptor activation (Kotani et al., 2001a; Ohtaki et al., 2001).

Alanine scanning mutagenesis and NMR studies on KP-10 demonstrated that the last five amino acids at the C-terminus—especially Phe^6^ and Arg^9^—are the primary determinants of the binding activity and the receptor activation potential of the decapeptide (Niida et al., 2006; Gutierrez-Pascual et al., 2009). NMR analysis also indicated that the C-terminal region of the decapeptide exhibits a helical structure in a simulated membrane environment, with specific amino acids, such as Asn^2^ and Phe^6^, located on the same side of the helix, which facilitates receptor binding (Lee et al., 2009; Curtis et al., 2010). These residues, together with the terminal aromatic groups (Phe^10^ and Tyr^10^) at the C-terminus, form the active center of KP-10, and their conservation across vertebrates underscores their essential role in receptor binding and activation (Orsini et al., 2007; Pasquier et al., 2014).

TAK-448 (MVT-602) is a synthetic KP analog originally designed to regulate the secretion of GnRH and reduce testosterone levels (Matsui et al., 2014). Its predecessor, TAK-683, was a shortened version of the KP-10 analog containing a series of modifications, including the introduction of D-tyrosine (d-Tyr), D-tryptophan (d-Trp), azaGly, and methylated arginine [Arg (Me)] (Asami et al., 2013). Both drugs (TAK-683 and TAK-448) are currently undergoing clinical trials to evaluate their potential in reproductive health disorders such as prostate cancer and polycystic ovary syndrome (PCOS) (Ishikawa et al., 2018; Tanaka et al., 2018; Abbara et al., 2020). However, TAK-683 exhibits limited water solubility and is prone to gel formation. To address this, researchers refined its structure by substituting the d-Trp at position 47 with a cyclic amino acid. This modification not only retains TAK-683’s potent agonistic activity but also markedly enhances its aqueous solubility, thus increasing its viability and potential in clinical applications (Nishizawa et al., 2016). In our recent work, we conducted a detailed study on the molecular basis of the interaction between TAK-448 and KISS1R, which is covered in subsequent sections (Hudson and Kauffman, 2022).

### Structure-based drug design for the optimization of arginine–phenylalanine–amide peptide analogs

SAR studies are a cornerstone of drug discovery, focusing on identifying key structural features of ligands that impact their biological activity. While SAR analysis excels in optimizing existing compounds, its utility is restricted when the precise 3D structure of a target protein is unknown, effectively limiting its capacity to optimize ligand-receptor interactions. Structure-Based Drug Design (SBDD) addresses this gap by using accurate receptor structures to directly inform ligand modifications, allowing for more refined and predictive drug design (Wang et al., 2018). Modern drug design often combines SBDD with SARS. By providing initial molecular structures through SBDD and optimizing the structural properties of these compounds using SAR analysis, researchers can more effectively design small molecule drugs with higher efficacy and lower side effects.

The advent of cryo-EM has revolutionized structure determination, providing unprecedented access to high-resolution structures of previously elusive protein targets, especially large or membrane-bound complexes such as GPCR-G protein complexes (Earl et al., 2017; Renaud et al., 2018; Shihoya et al., 2024). By capturing detailed receptor conformations in a near-native state, cryo-EM enables drug designers to visualize binding sites and conformational changes more clearly, which enhances both SBDD and SAR strategies. This integration of structural data allows for a more holistic understanding of the ligand-receptor dynamics and offers an invaluable foundation for rational drug design in complex biological systems.

**Figure 2:**
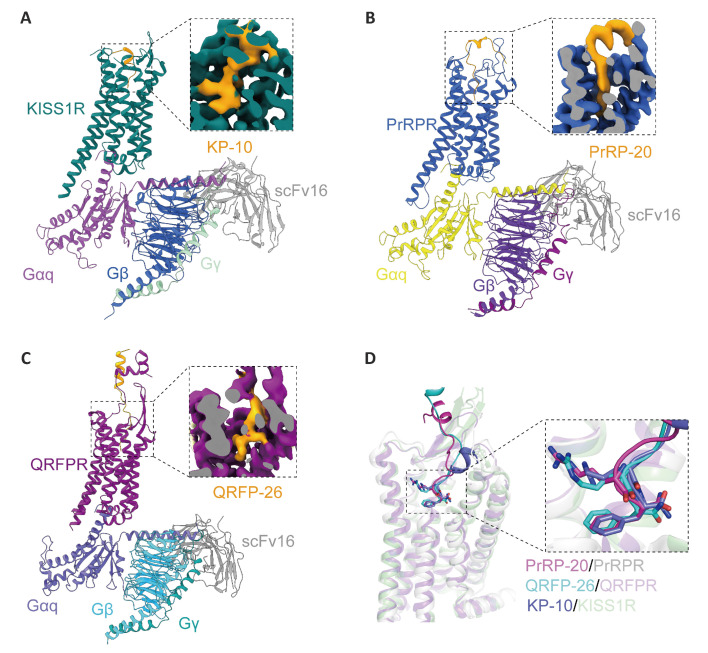
Cryo-electron microscopy structures of arginine–phenylalanine–amide receptor-G protein complexes. (A) KP10–KISS1R–G_q_–scFv16 complexes. (B) PrRP20–PrRPR–G_q_–scFv16 complexes. (C) QRFP26–QRFPR–G_q_–scFv16 complexes. (D) Binding pockets of KISS1R/PrRPR/QRFPR for their corresponding ligands. The molecular structure images were generated using PyMOL version 3.0 and UCSF ChimeraX version 1.6, and protein structure data were obtained from the PDB database (PDB IDs: 8XGS, 8ZPT, 8WZ2). KISS1R: Kisspeptin receptor; QRFP: pyroglutamylated arginine–phenylalanine–amide peptide; QRFPR: pyroglutamylated arginine–phenylalanine–amide peptide receptor.

The structures of KISS1R, PrRPR, and QRFPR complexed with their corresponding G proteins and ligands have been elucidated by cryo-EM (Hudson and Kauffman, 2022; Iwama et al., 2024; Jin et al., 2024; Li et al., 2024; **Figure 2**). These structures offer precise atomic-level insights invaluable for SBDD. Such molecular details facilitate the development of more selective and potent therapeutics, enhancing candidate binding efficiency and minimizing adverse effects.

#### Structural insights into prolactin-releasing peptide receptor and their significance for drug design

Li et al. (2024) were the first to resolve the cryo-EM structure of PrRP bound to PrRPR in complex with Gq or Gi heterotrimers. They identified a distinct pattern of PrRPR binding to PrRP20 (**Figure 2B** and **D**), characterized by an intricate network of polar and hydrophobic interactions between the receptor and the C-terminus of PrRP20. These findings provide a structural template for drug optimization as well as a basis for the design of selective PrRPR agonists or antagonists. By elucidating the differential coupling mechanisms between PrRPR and G_q_/G_i_ proteins, this study revealed the basis for selective G protein engagement (Li et al., 2024). These insights offer valuable guidance for the development of drugs that specifically activate desired signaling pathways by targeting specific receptor conformations.

#### Structural insights into pyroglutamylated arginine–phenylalanine–amide peptide receptor and their significance for drug design

Jin et al. (2024) resolved the cryo-EM structure of the G_q_ protein activated by the binding of 26RFa to QRFPR (**Figure 2C** and **D**). They identified a unique binding pattern between the N-terminus of 26RFa and the extracellular region of QRFPR, revealing how the C-terminal heptapeptide of 26RFa inserts into the transmembrane region of QRFPR, forming a broad interaction network. Their study provided molecular details of QRFPR-specific binding sites by elucidating how the C-terminal heptapeptide of 26RFa engages the transmembrane region of QRFPR. These findings enabled SBDD approaches aimed at optimizing the structure of small-molecule or peptide drugs in a targeted manner, thereby improving binding efficiency and selectivity. Meanwhile, they found that QRFPR activation follows a “message-address” pattern, in which the C-terminus of the peptide is responsible for activity and the N-terminus for receptor recognition (Jin et al., 2024). The elucidation of this mechanism provides a valuable structural basis for the development of agonists or antagonists with specific modes of action, thus helping to regulate receptor function in a targeted manner.

Iwama et al. (2024) described in detail the interaction between the C-terminal heptapeptide of 26RFa and the transmembrane domain of QRFPR, including hydrogen bonding and hydrophobic interaction networks. Building on cryo-EM structural data, they further analyzed in depth the conformational changes occurring in QRFPR when bound to 26RFa through molecular dynamics (MD) simulations. Their analysis revealed how transmembrane helices such as TM5 and TM6 undergo subtle movements during GPR103 receptor activation, contributing to a clearer understanding of the mechanism underlying this process (Iwama et al., 2024). This activation mechanism provides a structural foundation for designing drugs that can specifically activate or inhibit QRFPR, thus laying the groundwork for developing molecular tools capable of precisely modulating receptor function.

#### Structural insights into Kisspeptin receptor and their significance for drug design

Shen et al. (2024) resolved the cryo-EM structure of KISS1R binding to its endogenous agonist KP-10 (**Figure 2A** and **D**) and TAK-448, a synthetic analog of KP-10. They studied the conservative binding modes of KP-10 and TAK-448 and investigated the notable roles of key interacting residues, such as F195, N208, and W276, in KISS1R activation (Shen et al., 2024). These structural details can serve as the basis for designing novel KP-10 analogs or optimizing existing agonists (such as TAK-448), thus improving drug binding and selectivity.

Our work provides new insights into the diverse G protein coupling mechanisms and signaling pathway selectivity of KISSR1 (Hudson and Kauffman 2022). We found that KISS1R can couple with the G_i/o_ pathway in addition to the already known G_q/11_ pathway. This offers a fresh perspective for structure-based drug design (SBDD) targeting KISS1R, as different G protein couplings can lead to distinct physiological effects. This is especially promising for treatment, as drugs that can selectively activate or inhibit a specific pathway may have greater therapeutic potential. We also investigated the structural basis of Gi-coupled KISS1R when bound to TAK-448 (Hudson and Kauffman 2022). G_i_ pathway activation is related to a reduction in cAMP levels, which may influence glucose-stimulated insulin secretion (GSIS) and its pathological link to type 2 diabetes mellitus. Our structural data revealed conformational differences when KISS1R binds to G_q_ *versus* G_i_ proteins, providing richer structural information for the future design of KISS1R drugs that target specific signaling pathways. Therefore, this structural information can be used in SBDD to develop specific KISS1R agonists or antagonists for different signaling pathways.

#### Neuropeptide FF receptor ligand analog optimization based on homology modeling

Due to a range of complexities, the cryo-EM structures of NPFFRs remain unresolved. Despite this, Bender et al. (2023), employing a SBDD approach, constructed a homology model of NPFFR1, using structurally related proteins as templates to simulate its spatial conformation. Although this model has inherent limitations in structural precision, it nonetheless offered a sound structural framework for docking screens. Using this homology model as the docking platform, the authors identified candidate molecules with antagonistic properties. Subsequent structural optimization led to the development of a highly selective and potent NPFFR1 antagonist—compound 56—with a binding affinity of 22 nM.

Compound 56 significantly enhanced the analgesic efficacy of morphine while mitigating the common side effects associated with morphine monotherapy, such as constipation. Notably, it also exhibited intrinsic analgesic effects in murine models, even in the absence of morphine. These findings suggest that NPFFR1 antagonists can synergize with opioids, thereby enhancing analgesic effects while reducing opioid dosage and associated adverse effects.

## Limitations

This review has several limitations. Due to time constraints and the fast-paced development of the field, the most recent findings—particularly those involving high—resolution protein structures resolved by cryo-EM and advances in ligand design—could not be exhaustively covered. In addition, the signaling pathways downstream of the GPCRs discussed herein—especially those linking to specific disease mechanisms—remain only partially characterized and require further investigation. Finally, while preclinical data provide promising insights, clinical trial evidence remains limited, highlighting the need for continued translational studies to validate therapeutic relevance.

## Conclusion

Due to the pivotal role of RF-amide neuropeptide receptors in regulating a wide array of complex signaling pathways, this receptor family has emerged as a promising therapeutic target with significant potential in biological and biomedical research. The involvement of its members in essential physiological processes further underscores its value in drug development and highlights its potential to advance medical science.

The integration of SBDD and SAR analysis provides a comprehensive approach for developing drugs targeting RF-amide receptors, fostering innovations that could lead to more precise, effective, and safer therapies. This combination approach leverages structural and functional knowledge to refine drug design and potentially unlock novel therapeutic applications within this receptor family.

## Additional file:

***Additional Table 1:*** *Summaries of ligands, signaling pathways, distribution, and in vivo activity of RF-amide receptor family.*

## Data Availability

*All relevant data are within the paper and its Additional file*.

## References

[R1] Abbara A (2020). Kisspeptin receptor agonist has therapeutic potential for female reproductive disorders. J Clin Invest.

[R2] Adams C, Stroberg W, DeFazio RA, Schnell S, Moenter SM (2018). Gonadotropin-releasing hormone (GnRH) neuron excitability is regulated by estradiol feedback and kisspeptin. J Neurosci.

[R3] Alexopoulou F, Bech EM, Pedersen SL, Thorbek DD, Leurs U, Rudkjær LCB, Fosgerau K, Hansen HH, Vrang N, Jelsing J, Elster L (2022). Lipidated PrRP31 metabolites are long acting dual GPR10 and NPFF2 receptor agonists with potent body weight lowering effect. Sci Rep.

[R4] Alim K, Lefranc B, Sopkova-de Oliveira Santos J, Dubessy C, Picot M, Boutin JA, Vaudry H, Chartrel N, Vaudry D, Chuquet J, Leprince J (2018). Design, synthesis, molecular dynamics simulation, and functional evaluation of a novel series of 26RFa peptide analogues containing a mono- or polyalkyl guanidino arginine derivative. J Med Chem.

[R5] Anderson RA, Millar RP (2022). The roles of kisspeptin and neurokinin B in GnRH pulse generation in humans, and their potential clinical application. J Neuroendocrinol.

[R6] Aparicio SA (2005). Kisspeptins and GPR54--the new biology of the mammalian GnRH axis. Cell Metab.

[R7] Asami T, Nishizawa N, Matsui H, Nishibori K, Ishibashi Y, Horikoshi Y, Nakayama M, Matsumoto S, Tarui N, Yamaguchi M, Matsumoto H, Ohtaki T, Kitada C (2013). Design, synthesis, and biological evaluation of novel investigational nonapeptide KISS1R agonists with testosterone-suppressive activity. J Med Chem.

[R8] Aytürk N, Firat T, Kükner A, Özoğul C, Töre F, Kandirali İE, Yilmaz B (2017). The effect of kisspeptin on spermatogenesis and apoptosis in rats. Turk J Med Sci.

[R9] Bender BJ, Pickett JE, Braz J, Kang HJ, Gahbauer S, Bhardwaj K, Rodriguez-Rosado S, Liu Y, Jain M, Basbaum AI, Roth BL, Shoichet BK (2023). Structure-based discovery of a NPFF1R antagonist with analgesic activity. bioRxiv [Preprint].

[R10] Bihel F, Humbert JP, Schneider S, Bertin I, Wagner P, Schmitt M, Laboureyras E, Petit-Demoulière B, Schneider E, Mollereau C, Simonnet G, Simonin F, Bourguignon JJ (2015). Development of a peptidomimetic antagonist of neuropeptide FF receptors for the prevention of opioid-induced hyperalgesia. ACS Chem Neurosci.

[R11] Blasco V, Pinto FM, González-Ravina C, Santamaría-López E, Candenas L, Fernández-Sánchez M (2019). Tachykinins and kisspeptins in the regulation of human male fertility. J Clin Med.

[R12] Bonini JA (2000). Identification and characterization of two G protein-coupled receptors for neuropeptide FF. J Biol Chem.

[R13] Boyle RG, Downham R, Ganguly T, Humphries J, Smith J, Travers S (2005). Structure-activity studies on prolactin-releasing peptide (PrRP). Analogues of PrRP-(19-31)-peptide. J Pept Sci.

[R14] Brasnjevic I, Steinbusch HW, Schmitz C, Martinez-Martinez P, European NanoBioPharmaceutics Research Initiative (2009). Delivery of peptide and protein drugs over the blood-brain barrier. Prog Neurobiol.

[R15] Candelaria JI, Rabaglino MB, Denicol AC (2020). Ovarian preantral follicles are responsive to FSH as early as the primary stage of development. J Endocrinol.

[R16] Chan YM, Broder-Fingert S, Seminara SB (2009). Reproductive functions of kisspeptin and Gpr54 across the life cycle of mice and men. Peptides.

[R17] Chandel NS (2021). Lipid metabolism. Cold Spring Harb Perspect Biol.

[R18] Chartrel N, Picot M, El Medhi M, Arabo A, Berrahmoune H, Alexandre D, Maucotel J, Anouar Y, Prévost G (2016). The neuropeptide 26RFa (QRFP) and its role in the regulation of energy homeostasis: a mini-review. Front Neurosci.

[R19] Conrad P, Olenginski J, Nelson MD (2022). Exploring the role of redundant RFamide neuropeptide pathways during sleep in Caenorhabditis elegans. FASEB J.

[R20] Cook C, Nunn N, Worth AA, Bechtold DA, Suter T, Gackeheimer S, Foltz L, Emmerson PJ, Statnick MA, Luckman SM (2022). The hypothalamic RFamide, QRFP, increases feeding and locomotor activity: The role of Gpr103 and orexin receptors. PLoS One.

[R21] Curtis AE, Cooke JH, Baxter JE, Parkinson JR, Bataveljic A, Ghatei MA, Bloom SR, Murphy KG (2010). A kisspeptin-10 analog with greater in vivo bioactivity than kisspeptin-10. Am J Physiol Endocrinol Metab.

[R22] Czerwińska M, Czarzasta K, Cudnoch-Jędrzejewska A (2021). New peptides as potential players in the crosstalk between the brain and obesity, metabolic and cardiovascular diseases. Front Physiol.

[R23] Dai Y, Zhao X, Chen P, Yu Y, Wang Y, Xie L (2015). Neuropeptide FF promotes recovery of corneal nerve injury associated with hyperglycemia. Invest Ophthalmol Vis Sci.

[R24] Davis XS, Grill HJ (2018). The hindbrain is a site of energy balance action for prolactin-releasing peptide: feeding and thermic effects from GPR10 stimulation of the nucleus tractus solitarius/area postrema. Psychopharmacology (Berl).

[R25] Devère M, Takhlidjt S, Prévost G, Chartrel N, Leprince J, Picot M (2025). The 26RFa (QRFP)/GPR103 neuropeptidergic system: a key regulator of energy and glucose metabolism. Neuroendocrinology.

[R26] do Rego JC, Leprince J, Chartrel N, Vaudry H, Costentin J (2006). Behavioral effects of 26RFamide and related peptides. Peptides.

[R27] Dockray GJ (2004). The expanding family of -RFamide peptides and their effects on feeding behaviour. Exp Physiol.

[R28] Drieu la Rochelle A, Guillemyn K, Dumitrascuta M, Martin C, Utard V, Quillet R, Schneider S, Daubeuf F, Willemse T, Mampuys P, Maes BUW, Frossard N, Bihel F, Spetea M, Simonin F, Ballet S (2018). A bifunctional-biased mu-opioid agonist-neuropeptide FF receptor antagonist as analgesic with improved acute and chronic side effects. Pain.

[R29] Duan J, Shen DD, Zhao T, Guo S, He X, Yin W, Xu P, Ji Y, Chen LN, Liu J, Zhang H, Liu Q, Shi Y, Cheng X, Jiang H, Eric Xu H, Zhang Y, Xie X, Jiang Y (2022). Molecular basis for allosteric agonism and G protein subtype selectivity of galanin receptors. Nat Commun.

[R30] D’Ursi AM, Albrizio S, Di Fenza A, Crescenzi O, Carotenuto A, Picone D, Novellino E, Rovero P (2002). Structural studies on Hgr3 orphan receptor ligand prolactin-releasing peptide. J Med Chem.

[R31] Dwyer AA, Quinton R, Llahana S, Follin C, Yedinak C, Grossman A (2019). Anatomy and physiology of the hypothalamic-pituitary-gonadal (HPG) axis. Advanced practice in endocrinology nursing.

[R32] Earl LA, Falconieri V, Milne JL, Subramaniam S (2017). Cryo-EM: beyond the microscope. Curr Opin Struct Biol.

[R33] El Mehdi M, Takhlidjt S, Devère M, Arabo A, Le Solliec MA, Maucotel J, Bénani A, Nedelec E, Duparc C, Lefranc B, Leprince J, Anouar Y, Prévost G, Chartrel N, Picot M (2022). The 26RFa (QRFP)/GPR103 neuropeptidergic system in mice relays insulin signalling into the brain to regulate glucose homeostasis. Diabetologia.

[R34] Elhabazi K (2017). RF313, an orally bioavailable neuropeptide FF receptor antagonist, opposes effects of RF-amide-related peptide-3 and opioid-induced hyperalgesia in rodents. Neuropharmacology.

[R35] Elshourbagy NA, Ames RS, Fitzgerald LR, Foley JJ, Chambers JK, Szekeres PG, Evans NA, Schmidt DB, Buckley PT, Dytko GM, Murdock PR, Milligan G, Groarke DA, Tan KB, Shabon U, Nuthulaganti P, Wang DY, Wilson S, Bergsma DJ, Sarau HM (2000). Receptor for the pain modulatory neuropeptides FF and AF is an orphan G protein-coupled receptor. J Biol Chem.

[R36] Engström M, Brandt A, Wurster S, Savola JM, Panula P (2003). Prolactin releasing peptide has high affinity and efficacy at neuropeptide FF2 receptors. J Pharmacol Exp Ther.

[R37] Fang Q, Liu Q, Li N, Jiang TN, Li YL, Yan X, Wang R (2009). Cardiovascular effects of intravenous administered 26RFa, a novel RFamide peptide ligand for GPR103, in anaesthetised rats. Eur J Pharmacol.

[R38] Findeisen M, Rathmann D, Beck-Sickinger AG (2011). RFamide peptides: structure, function, mechanisms and pharmaceutical potential. Pharmaceuticals.

[R39] Fukusumi S, Yoshida H, Fujii R, Maruyama M, Komatsu H, Habata Y, Shintani Y, Hinuma S, Fujino M (2003). A new peptidic ligand and its receptor regulating adrenal function in rats. J Biol Chem.

[R40] Fuqua JS, Eugster EA (2022). History of puberty: normal and precocious. Horm Res Paediatr.

[R41] Garcia JP, Keen KL, Kenealy BP, Seminara SB, Terasawa E (2018). Role of kisspeptin and neurokinin B signaling in male rhesus monkey puberty. Endocrinology.

[R42] García-Ortega J, Pinto FM, Fernández-Sánchez M, Prados N, Cejudo-Román A, Almeida TA, Hernández M, Romero M, Tena-Sempere M, Candenas L (2014). Expression of neurokinin B/NK3 receptor and kisspeptin/KISS1 receptor in human granulosa cells. Hum Reprod.

[R43] Gaytan F, Garcia-Galiano D, Dorfman MD, Manfredi-Lozano M, Castellano JM, Dissen GA, Ojeda SR, Tena-Sempere M (2014). Kisspeptin receptor haplo-insufficiency causes premature ovarian failure despite preserved gonadotropin secretion. Endocrinology.

[R44] Georgsson J, Bergström F, Nordqvist A, Watson MJ, Blundell CD, Johansson MJ, Petersson AU, Yuan ZQ, Zhou Y, Kristensson L, Kakol-Palm D, Tyrchan C, Wellner E, Bauer U, Brodin P, Svensson Henriksson A (2014). GPR103 antagonists demonstrating anorexigenic activity in vivo: design and development of pyrrolo[2,3-c]pyridines that mimic the C-terminal Arg-Phe motif of QRFP26. J Med Chem.

[R45] Gicquel S, Mazarguil H, Allard M, Simonnet G, Zajac JM (1992). Analogues of F8Famide resistant to degradation, with high affinity and in vivo effects. Eur J Pharmacol.

[R46] Gicquel S, Mazarguil H, Desprat C, Allard M, Devillers JP, Simonnet G, Zajac JM (1994). Structure-activity study of neuropeptide FF: contribution of N-terminal regions to affinity and activity. J Med Chem.

[R47] Gołyszny M, Obuchowicz E, Zieliński M (2022). Neuropeptides as regulators of the hypothalamus-pituitary-gonadal (HPG) axis activity and their putative roles in stress-induced fertility disorders. Neuropeptides.

[R48] Goncharuk V, Zeng Z, Wang R, MacTavish D, Jhamandas JH (2004). Distribution of the neuropeptide FF1 receptor (hFF1) in the human hypothalamus and surrounding basal forebrain structures: immunohistochemical study. J Comp Neurol.

[R49] Goncharuk V, Jhamandas JH (2008). Neuropeptide FF2 receptor distribution in the human brain: An immunohistochemical study. Peptides.

[R50] Gouardères C, Jhamandas K, Sutak M, Zajac JM (1996). Role of opioid receptors in the spinal antinociceptive effects of neuropeptide FF analogues. Br J Pharmacol.

[R51] Gouardères C, Tafani JA, Zajac JM (1998). Affinity of neuropeptide FF analogs to opioid receptors in the rat spinal cord. Peptides.

[R52] Gouardères C, Mazarguil H, Mollereau C, Chartrel N, Leprince J, Vaudry H, Zajac JM (2007). Functional differences between NPFF1 and NPFF2 receptor coupling: high intrinsic activities of RFamide-related peptides on stimulation of [35S]GTPgammaS binding. Neuropharmacology.

[R53] Granata R, Settanni F, Trovato L, Gallo D, Gesmundo I, Nano R, Gallo MP, Bergandi L, Volante M, Alloatti G, Piemonti L, Leprince J, Papotti M, Vaudry H, Ong H, Ghigo E (2014). RFamide peptides 43RFa and 26RFa both promote survival of pancreatic β-cells and human pancreatic islets but exert opposite effects on insulin secretion. Diabetes.

[R54] Guha R (2013). On exploring structure-activity relationships. Methods Mol Biol.

[R55] Gutiérrez-Pascual E, Leprince J, Martínez-Fuentes AJ, Ségalas-Milazzo I, Pineda R, Roa J, Duran-Prado M, Guilhaudis L, Desperrois E, Lebreton A, Pinilla L, Tonon MC, Malagón MM, Vaudry H, Tena-Sempere M, Castaño JP (2009). In vivo and in vitro structure-activity relationships and structural conformation of Kisspeptin-10-related peptides. Mol Pharmacol.

[R56] Guzman S (2022). Targeting hepatic kisspeptin receptor ameliorates nonalcoholic fatty liver disease in a mouse model. J Clin Invest.

[R57] Han SK, Gottsch ML, Lee KJ, Popa SM, Smith JT, Jakawich SK, Clifton DK, Steiner RA, Herbison AE (2005). Activation of gonadotropin-releasing hormone neurons by kisspeptin as a neuroendocrine switch for the onset of puberty. J Neurosci.

[R58] Hinuma S, Habata Y, Fujii R, Kawamata Y, Hosoya M, Fukusumi S, Kitada C, Masuo Y, Asano T, Matsumoto H, Sekiguchi M, Kurokawa T, Nishimura O, Onda H, Fujino M (1998). A prolactin-releasing peptide in the brain. Nature.

[R59] Hökfelt T, Broberger C, Xu ZQ, Sergeyev V, Ubink R, Diez M (2000). Neuropeptides--an overview. Neuropharmacology.

[R60] Holt MK, Rinaman L (2022). The role of nucleus of the solitary tract glucagon-like peptide-1 and prolactin-releasing peptide neurons in stress: anatomy, physiology and cellular interactions. Br J Pharmacol.

[R61] Hrabovszky E, Ciofi P, Vida B, Horvath MC, Keller E, Caraty A, Bloom SR, Ghatei MA, Dhillo WS, Liposits Z, Kallo I (2010). The kisspeptin system of the human hypothalamus: sexual dimorphism and relationship with gonadotropin-releasing hormone and neurokinin B neurons. Eur J Neurosci.

[R62] Hudson AD, Kauffman AS (2022). Metabolic actions of kisspeptin signaling: Effects on body weight, energy expenditure, and feeding. Pharmacol Ther.

[R63] Hunt SC, Hasstedt SJ, Xin Y, Dalley BK, Milash BA, Yakobson E, Gress RE, Davidson LE, Adams TD (2011). Polymorphisms in the NPY2R gene show significant associations with BMI that are additive to FTO, MC4R, and NPFFR2 gene effects. Obesity (Silver Spring).

[R64] Ibata Y, Iijima N, Kataoka Y, Kakihara K, Tanaka M, Hosoya M, Hinuma S (2000). Morphological survey of prolactin-releasing peptide and its receptor with special reference to their functional roles in the brain. Neurosci Res.

[R65] Ikegami K, Goto T, Nakamura S, Watanabe Y, Sugimoto A, Majarune S, Horihata K, Nagae M, Tomikawa J, Imamura T, Sanbo M, Hirabayashi M, Inoue N, Maeda KI, Tsukamura H, Uenoyama Y (2020). Conditional kisspeptin neuron-specific Kiss1 knockout with newly generated Kiss1-floxed and Kiss1-Cre mice replicates a hypogonadal phenotype of global Kiss1 knockout mice. J Reprod Dev.

[R66] Ilahi I, Haq TU (2021). MINI REVIEW: Role of Kisspeptin-GPR54 system in regulation of reproductive functions in human and other mammals. Pak J Pharm Sci.

[R67] Ishikawa K, Tanaka A, Kogame A, Watanabe T, Tagawa Y, Matsui H (2018). Usefulness of pharmacokinetic/efficacy analysis of an investigational kisspeptin analog, TAK–448, in quantitatively evaluating anti-tumor growth effect in the rat VCaP androgen-sensitive prostate cancer model. Eur J Pharmacol.

[R68] Iwama A, Kise R, Akasaka H, Sano FK, Oshima HS, Inoue A, Shihoya W, Nureki O (2024). Structure and dynamics of the pyroglutamylated RF-amide peptide QRFP receptor GPR103. Nat Commun.

[R69] Jiang Y, Luo L, Gustafson EL, Yadav D, Laverty M, Murgolo N, Vassileva G, Zeng M, Laz TM, Behan J, Qiu P, Wang L, Wang S, Bayne M, Greene J, Monsma F, Zhang FL (2003). Identification and characterization of a novel RF-amide peptide ligand for orphan G-protein-coupled receptor SP9155. J Biol Chem.

[R70] Jin S, Guo S, Xu Y, Li X, Wu C, He X, Pan B, Xin W, Zhang H, Hu W, Yin Y, Zhang T, Wu K, Yuan Q, Xu HE, Xie X, Jiang Y (2024). Structural basis for recognition of 26RFa by the pyroglutamylated RFamide peptide receptor. Cell Discov.

[R71] Jörgensen SK, Karnošová A, Mazzaferro S, Rowley O, Chen HC, Robbins SJ, Christofides S, Merkle FT, Maletínská L, Petrik D (2024). An analogue of the Prolactin Releasing Peptide reduces obesity and promotes adult neurogenesis. EMBO Rep.

[R72] Kalliomäki ML, Pertovaara A, Brandt A, Wei H, Pietilä P, Kalmari J, Xu M, Kalso E, Panula P (2004). Prolactin-releasing peptide affects pain, allodynia and autonomic reflexes through medullary mechanisms. Neuropharmacology.

[R73] Karnošová A, Strnadová V, Holá L, Železná B, Kuneš J, Maletínská L (2021). Palmitoylation of prolactin-releasing peptide increased affinity for and activation of the GPR10, NPFF–R2 and NPFF-R1 receptors: in vitro study. Int J Mol Sci.

[R74] Kawakami A, Okada N, Rokkaku K, Honda K, Ishibashi S, Onaka T (2008). Leptin inhibits and ghrelin augments hypothalamic noradrenaline release after stress. Stress.

[R75] Keen KL, Wegner FH, Bloom SR, Ghatei MA, Terasawa E (2008). An increase in kisspeptin-54 release occurs with the pubertal increase in luteinizing hormone-releasing hormone-1 release in the stalk-median eminence of female rhesus monkeys in vivo. Endocrinology.

[R76] Kenakin T (2019). Can we use nature’s structure-activity-relationships to make better drugs?. Drug Discov Today.

[R77] Khan HL, Bhatti S, Sehole Z, Younas H, Nathaniel S, Abbas S, Kaloglu C, Ziders R, Yildiz A, Isa AM (2022). Putative role of the Kisspeptin/Kiss1R system in promoting hypothalamic GnRH release, pubertal maturation, and regulation of ovulation considering the central reproductive axis. Reprod Fertil.

[R78] Khan SH, Chaudhry N (2021). Beyond GnRH, LH and FSH: The role of kisspeptin on hypothalalmic-pituitary gonadal (HPG) axis pathology and diagnostic consideration. J Pak Med Assoc.

[R79] Kim TH, Yoon JH, Cho SG (2020). Kisspeptin promotes glioblastoma cell invasiveness via the Gq-PLC-PKC pathway. Anticancer Res.

[R80] Kimura A, Ohmichi M, Tasaka K, Kanda Y, Ikegami H, Hayakawa J, Hisamoto K, Morishige K, Hinuma S, Kurachi H, Murata Y (2000). Prolactin-releasing peptide activation of the prolactin promoter is differentially mediated by extracellular signal-regulated protein kinase and c-Jun N-terminal protein kinase. J Biol Chem.

[R81] Kotani M, Detheux M, Vandenbogaerde A, Communi D, Vanderwinden JM, Le Poul E, Brézillon S, Tyldesley R, Suarez-Huerta N, Vandeput F, Blanpain C, Schiffmann SN, Vassart G, Parmentier M (2001). The metastasis suppressor gene KiSS-1 encodes kisspeptins, the natural ligands of the orphan G protein-coupled receptor GPR54. J Biol Chem.

[R82] Kotani M, Mollereau C, Detheux M, Le Poul E, Brézillon S, Vakili J, Mazarguil H, Vassart G, Zajac JM, Parmentier M (2001). Functional characterization of a human receptor for neuropeptide FF and related peptides. Br J Pharmacol.

[R83] Kovács A, Szabó E, László K, Kertes E, Zagorácz O, Mintál K, Tóth A, Gálosi R, Berta B, Lénárd L, Hormay E, László B, Zelena D, Tóth ZE (2024). Brain RFamide neuropeptides in stress-related psychopathologies. Cells.

[R84] Lawrence CB, Celsi F, Brennand J, Luckman SM (2000). Alternative role for prolactin-releasing peptide in the regulation of food intake. Nat Neurosci.

[R85] Lawrence CB, Ellacott KL, Luckman SM (2002). PRL-releasing peptide reduces food intake and may mediate satiety signaling. Endocrinology.

[R86] Le Solliec MA, Arabo A, Takhlidjt S, Maucotel J, Devère M, Riancho J, Berrahmoune H, do Rego JL, do Rego JC, Bénani A, Nedelec E, Lefranc B, Leprince J, Anouar Y, Picot M, Chartrel N, Prévost G (2022). Acute but not chronic central administration of the neuropeptide 26RFa (QRFP) improves glucose homeostasis in obese/diabetic mice. Neuroendocrinology.

[R87] Lee DA, Andreev A, Truong TV, Chen A, Hill AJ, Oikonomou G, Pham U, Hong YK, Tran S, Glass L, Sapin V, Engle J, Fraser SE, Prober DA (2017). Genetic and neuronal regulation of sleep by neuropeptide VF. Elife.

[R88] Lee DK, Nguyen T, O’Neill GP, Cheng R, Liu Y, Howard AD, Coulombe N, Tan CP, Tang-Nguyen AT, George SR, O’Dowd BF (1999). Discovery of a receptor related to the galanin receptors. FEBS Lett.

[R89] Lee JH, Miele ME, Hicks DJ, Phillips KK, Trent JM, Weissman BE, Welch DR (1996). KiSS-1, a novel human malignant melanoma metastasis-suppressor gene. J Natl Cancer Inst.

[R90] Lee JY, Moon JS, Eu YJ, Lee CW, Yang ST, Lee SK, Jung HH, Kim HH, Rhim H, Seong JY, Kim JI (2009). Molecular interaction between kisspeptin decapeptide analogs and a lipid membrane. Arch Biochem Biophys.

[R91] Lefranc B, Alim K, Neveu C, Le Marec O, Dubessy C, Boutin JA, Chuquet J, Vaudry D, Prévost G, Picot M, Vaudry H, Chartrel N, Leprince J (2021). Point-substitution of phenylalanine residues of 26RFa neuropeptide: a structure-activity relationship study. Molecules.

[R92] Leprince J, Bagnol D, Bureau R, Fukusumi S, Granata R, Hinuma S, Larhammar D, Primeaux S, Sopkova-de Oliveiras Santos J, Tsutsui K, Ukena K, Vaudry H (2017). The Arg-Phe-amide peptide 26RFa/glutamine RF-amide peptide and its receptor: IUPHAR Review 24. Br J Pharmacol.

[R93] Li N, Han ZL, Wang ZL, Xing YH, Sun YL, Li XH, Song JJ, Zhang T, Zhang R, Zhang MN, Xu B, Fang Q, Wang R (2016). BN-9, a chimeric peptide with mixed opioid and neuropeptide FF receptor agonistic properties, produces nontolerance-forming antinociception in mice. Br J Pharmacol.

[R94] Li Y, Yuan Q, He X, Zhang Y, You C, Wu C, Li J, Xu HE, Zhao LH (2024). Molecular mechanism of prolactin-releasing peptide recognition and signaling via its G protein-coupled receptor. Cell Discov.

[R95] Lin SH, Leslie FM, Civelli O (2002). Neurochemical properties of the prolactin releasing peptide (PrRP) receptor expressing neurons: evidence for a role of PrRP as a regulator of stress and nociception. Brain Res.

[R96] Lin YT, Yu YL, Hong WC, Yeh TS, Chen TC, Chen JC (2017). NPFFR2 activates the HPA axis and induces anxiogenic effects in rodents. Int J Mol Sci.

[R97] Lin YT, Chen JC (2019). Neuropeptide FF modulates neuroendocrine and energy homeostasis through hypothalamic signaling. Chin J Physiol.

[R98] Liu X, Herbison AE (2014). RF9 excitation of GnRH neurons is dependent upon Kiss1r in the adult male and female mouse. Endocrinology.

[R99] Ma Q, Cao Z, Li H, Wang W, Tian Y, Yan L, Liao Y, Chen X, Chen Y, Shi Y, Tang S, Zhou N (2021). Two naturally occurring mutations of human GPR103 define distinct G protein selection bias. Biochim Biophys Acta Mol Cell Res.

[R100] Maletínská L, Spolcová A, Maixnerová J, Blechová M, Zelezná B (2011). Biological properties of prolactin-releasing peptide analogs with a modified aromatic ring of a C-terminal phenylalanine amide. Peptides.

[R101] Maletínská L, Tichá A, Nagelová V, Spolcová A, Blechová M, Elbert T, Zelezná B (2013). Neuropeptide FF analog RF9 is not an antagonist of NPFF receptor and decreases food intake in mice after its central and peripheral administration. Brain Res.

[R102] Maletínská L, Nagelová V, Tichá A, Zemenová J, Pirník Z, Holubová M, Špolcová A, Mikulášková B, Blechová M, Sýkora D, Lacinová Z, Haluzík M, Železná B, Kuneš J (2015). Novel lipidized analogs of prolactin-releasing peptide have prolonged half-lives and exert anti-obesity effects after peripheral administration. Int J Obes (Lond).

[R103] Mamgain A, Sawyer IL, Timajo DAM, Rizwan MZ, Evans MC, Ancel CM, Inglis MA, Anderson GM (2021). RFamide-related peptide neurons modulate reproductive function and stress responses. J Neurosci.

[R104] Maniscalco JW, Rinaman L (2017). Interoceptive modulation of neuroendocrine, emotional, and hypophagic responses to stress. Physiol Behav.

[R105] Marchese A (1995). Cloning and chromosomal mapping of three novel genes, GPR9, GPR10, and GPR14, encoding receptors related to interleukin 8, neuropeptide Y, and somatostatin receptors. Genomics.

[R106] Matsui H, Masaki T, Akinaga Y, Kiba A, Takatsu Y, Nakata D, Tanaka A, Ban J, Matsumoto S, Kumano S, Suzuki A, Ikeda Y, Yamaguchi M, Watanabe T, Ohtaki T, Kusaka M (2014). Pharmacologic profiles of investigational kisspeptin/metastin analogues, TAK–448 and TAK-683, in adult male rats in comparison to the GnRH analogue leuprolide. Eur J Pharmacol.

[R107] Medeiros SF, Barbosa BB, Medeiros MAS, Yamamoto MMW (2021). Morphology and biochemistry of ovulation. Rev Bras Ginecol Obstet.

[R108] Mei H, Doran J, Kyle V, Yeo SH, Colledge WH (2013). Does kisspeptin signaling have a role in the testes?. Front Endocrinol (Lausanne).

[R109] Moenter SM, Evans NP (2022). Gonadotropin-releasing hormone (GnRH) measurements in pituitary portal blood: A history. J Neuroendocrinol.

[R110] Mollereau C, Mazarguil H, Zajac JM, Roumy M (2005). Neuropeptide FF (NPFF) analogs functionally antagonize opioid activities in NPFF2 receptor-transfected SH-SY5Y neuroblastoma cells. Mol Pharmacol.

[R111] Morales T, Sawchenko PE (2003). Brainstem prolactin-releasing peptide neurons are sensitive to stress and lactation. Neuroscience.

[R112] Morgan A, Shekhar N, Strnadová V, Pirník Z, Haasová E, Kopecký J, Pačesová A, Železná B, Kuneš J, Bardová K, Maletínská L (2024). Deficiency of GPR10 and NPFFR2 receptors leads to sex-specific prediabetic syndrome and late-onset obesity in mice. Biosci Rep.

[R113] Muir AI (2001). AXOR12, a novel human G protein-coupled receptor, activated by the peptide KiSS-1. J Biol Chem.

[R114] Nash KT, Welch DR (2006). The KISS1 metastasis suppressor: mechanistic insights and clinical utility. Front Biosci.

[R115] Navarro VM, Fernández-Fernández R, Castellano JM, Roa J, Mayen A, Barreiro ML, Gaytan F, Aguilar E, Pinilla L, Dieguez C, Tena-Sempere M (2004). Advanced vaginal opening and precocious activation of the reproductive axis by KiSS-1 peptide, the endogenous ligand of GPR54. J Physiol.

[R116] Navarro VM, Fernández-Fernández R, Nogueiras R, Vigo E, Tovar S, Chartrel N, Le Marec O, Leprince J, Aguilar E, Pinilla L, Dieguez C, Vaudry H, Tena-Sempere M (2006). Novel role of 26RFa, a hypothalamic RFamide orexigenic peptide, as putative regulator of the gonadotropic axis. J Physiol.

[R117] Navarro VM (2020). Metabolic regulation of kisspeptin - the link between energy balance and reproduction. Nat Rev Endocrinol.

[R118] Nguyen T, Marusich J, Li JX, Zhang Y (2020). Neuropeptide FF and its receptors: therapeutic applications and ligand development. J Med Chem.

[R119] Nichols R, Bass C, Demers L, Larsen B, Li E, Blewett N, Converso-Baran K, Russell MW, Westfall MV (2012). Structure-activity studies of RFamide-related peptide-1 identify a functional receptor antagonist and novel cardiac myocyte signaling pathway involved in contractile performance. J Med Chem.

[R120] Nieminen ML, Nystedt J, Panula P (2003). Expression of neuropeptide FF, prolactin-releasing peptide, and the receptor UHR1/GPR10 genes during embryogenesis in the rat. Dev Dyn.

[R121] Niida A, Wang Z, Tomita K, Oishi S, Tamamura H, Otaka A, Navenot JM, Broach JR, Peiper SC, Fujii N (2006). Design and synthesis of downsized metastin (45-54) analogs with maintenance of high GPR54 agonistic activity. Bioorg Med Chem Lett.

[R122] Nishizawa N, Takatsu Y, Kumano S, Kiba A, Ban J, Tsutsumi S, Matsui H, Matsumoto SI, Yamaguchi M, Ikeda Y, Kusaka M, Ohtaki T, Itoh F, Asami T (2016). Design and synthesis of an investigational nonapeptide kiss1 receptor (KISS1R) agonist, Ac-d-Tyr-hydroxyproline (Hyp)-Asn-Thr-Phe-azaGly-Leu-Arg(Me)-Trp-NH2 (TAK-448), with highly potent testosterone-suppressive activity and excellent water solubility. J Med Chem.

[R123] Nourbakhsh F, Atabaki R, Roohbakhsh A (2018). The role of orphan G protein-coupled receptors in the modulation of pain: A review. Life Sci.

[R124] Ohtaki T (2001). Metastasis suppressor gene KiSS-1 encodes peptide ligand of a G-protein-coupled receptor. Nature.

[R125] Oishi S, Misu R, Tomita K, Setsuda S, Masuda R, Ohno H, Naniwa Y, Ieda N, Inoue N, Ohkura S, Uenoyama Y, Tsukamura H, Maeda K, Hirasawa A, Tsujimoto G, Fujii N (2010). Activation of neuropeptide FF receptors by kisspeptin receptor ligands. ACS Med Chem Lett.

[R126] Onaka T, Takayanagi Y, Leng G (2010). Metabolic and stress-related roles of prolactin-releasing peptide. Trends Endocrinol Metab.

[R127] Orsini MJ, Klein MA, Beavers MP, Connolly PJ, Middleton SA, Mayo KH (2007). Metastin (KiSS-1) mimetics identified from peptide structure-activity relationship-derived pharmacophores and directed small molecule database screening. J Med Chem.

[R128] Owen CM, Zhou X, Bernard DJ, Jaffe LA (2021). Kisspeptin-54 injection induces a physiological luteinizing hormone surge and ovulation in mice. Biol Reprod.

[R129] Palkovits M, Záborszky L, Feminger A, Mezey E, Fekete MI, Herman JP, Kanyicska B, Szabó D (1980). Noradrenergic innervation of the rat hypothalamus:experimental biochemical and electron microscopic studies. Brain Res.

[R130] Pasquier J, Kamech N, Lafont AG, Vaudry H, Rousseau K, Dufour S (2014). Molecular evolution of GPCRs: Kisspeptin/kisspeptin receptors. J Mol Endocrinol.

[R131] Pražienková V, Holubová M, Pelantová H, Bugáňová M, Pirník Z, Mikulášková B, Popelová A, Blechová M, Haluzík M, Železná B, Kuzma M, Kuneš J, Maletínská L (2017). Impact of novel palmitoylated prolactin-releasing peptide analogs on metabolic changes in mice with diet-induced obesity. PLoS One.

[R132] Pražienková V, Popelová A, Kuneš J, Maletínská L (2019). Prolactin-releasing peptide: physiological and pharmacological properties. Int J Mol Sci.

[R133] Pražienková V, Funda J, Pirník Z, Karnošová A, Hrubá L, Kořínková L, Neprašová B, Janovská P, Benzce M, Kadlecová M, Blahoš J, Kopecký J, Železná B, Kuneš J, Bardová K, Maletínská L (2021). GPR10 gene deletion in mice increases basal neuronal activity, disturbs insulin sensitivity and alters lipid homeostasis. Gene.

[R134] Prévost G, Jeandel L, Arabo A, Coëffier M, El Ouahli M, Picot M, Alexandre D, Gobet F, Leprince J, Berrahmoune H, Déchelotte P, Malagon M, Bonner C, Kerr-Conte J, Chigr F, Lefebvre H, Anouar Y, Chartrel N (2015). Hypothalamic neuropeptide 26RFa acts as an incretin to regulate glucose homeostasis. Diabetes.

[R135] Prévost G, Picot M, Le Solliec MA, Arabo A, Berrahmoune H, El Mehdi M, Cherifi S, Benani A, Nédélec E, Gobet F, Brunel V, Leprince J, Lefebvre H, Anouar Y, Chartrel N (2019). The neuropeptide 26RFa in the human gut and pancreas: potential involvement in glucose homeostasis. Endocr Connect.

[R136] Quillet R, Ayachi S, Bihel F, Elhabazi K, Ilien B, Simonin F (2016). RF-amide neuropeptides and their receptors in Mammals: Pharmacological properties, drug development and main physiological functions. Pharmacol Ther.

[R137] Raffa RB (1988). The action of FMRFamide (Phe-Met-Arg-Phe-NH2) and related peptides on mammals. Peptides.

[R138] Renaud JP, Chari A, Ciferri C, Liu WT, Rémigy HW, Stark H, Wiesmann C (2018). Cryo-EM in drug discovery: achievements, limitations and prospects. Nat Rev Drug Discov.

[R139] Roger C, Lasbleiz A, Guye M, Dutour A, Gaborit B, Ranjeva JP (2022). The Role of the Human Hypothalamus in Food Intake Networks: An MRI Perspective. Front Nutr.

[R140] Roky R, Obál F, Valatx JL, Bredow S, Fang J, Pagano LP, Krueger JM (1995). Prolactin and rapid eye movement sleep regulation. Sleep.

[R141] Roland BL, Sutton SW, Wilson SJ, Luo L, Pyati J, Huvar R, Erlander MG, Lovenberg TW (1999). Anatomical distribution of prolactin-releasing peptide and its receptor suggests additional functions in the central nervous system and periphery. Endocrinology.

[R142] Rosenbaum DM, Rasmussen SG, Kobilka BK (2009). The structure and function of G-protein-coupled receptors. Nature.

[R143] Seal LJ, Small CJ, Dhillo WS, Kennedy AR, Ghatei MA, Bloom SR (2002). Prolactin-releasing peptide releases corticotropin-releasing hormone and increases plasma adrenocorticotropin via the paraventricular nucleus of the hypothalamus. Neuroendocrinology.

[R144] Shahab M, Mastronardi C, Seminara SB, Crowley WF, Ojeda SR, Plant TM (2005). Increased hypothalamic GPR54 signaling: a potential mechanism for initiation of puberty in primates. Proc Natl Acad Sci U S A.

[R145] Shen S, Wang D, Liu H, He X, Cao Y, Chen J, Li S, Cheng X, Xu HE, Duan J (2024). Structural basis for hormone recognition and distinctive Gq protein coupling by the kisspeptin receptor. Cell Rep.

[R146] Shihoya W, Iwama A, Sano FK, Nureki O (2024). Cryo-EM advances in GPCR structure determination. J Biochem.

[R147] Shin Y, Jung W, Kim MY, Shin D, Kim GH, Kim CH, Park SH, Cho EH, Choi DW, Han CJ, Lee KH, Kim SB, Shin HJ (2022). NPFFR2 contributes to the malignancy of hepatocellular carcinoma development by activating RhoA/YAP signaling. Cancers (Basel).

[R148] Silva PHAD, Romão LGM, Freitas NPA, Carvalho TR, Porto MEMP, Araujo E, Cavalcante MB (2023). Kisspeptin as a predictor of miscarriage: a systematic review. J Matern Fetal Neonatal Med.

[R149] Simonin F, Schmitt M, Laulin JP, Laboureyras E, Jhamandas JH, MacTavish D, Matifas A, Mollereau C, Laurent P, Parmentier M, Kieffer BL, Bourguignon JJ, Simonnet G (2006). RF9, a potent and selective neuropeptide FF receptor antagonist, prevents opioid-induced tolerance associated with hyperalgesia. Proc Natl Acad Sci U S A.

[R150] Song WJ, Mondal P, Wolfe A, Alonso LC, Stamateris R, Ong BW, Lim OC, Yang KS, Radovick S, Novaira HJ, Farber EA, Farber CR, Turner SD, Hussain MA (2014). Glucagon regulates hepatic kisspeptin to impair insulin secretion. Cell Metab.

[R151] Steriade M (2005). Sleep, epilepsy and thalamic reticular inhibitory neurons. Trends Neurosci.

[R152] Stevenson H, Bartram S, Charalambides MM, Murthy S, Petitt T, Pradeep A, Vineall O, Abaraonye I, Lancaster A, Koysombat K, Patel B, Abbara A (2022). Kisspeptin-neuron control of LH pulsatility and ovulation. Front Endocrinol (Lausanne).

[R153] Strnadová V, Morgan A, Škrlová M, Haasová E, Bardová K, Myšková A, Sýkora D, Kuneš J, Železná B, Maletínská L (2024). Peripheral administration of lipidized NPAF and NPFF analogs does not influence central food intake regulation but induces anxiety-like behavior. Neuropeptides.

[R154] Sullivan-Pyke C, Haisenleder DJ, Senapati S, Nicolais O, Eisenberg E, Sammel MD, Barnhart KT (2018). Kisspeptin as a new serum biomarker to discriminate miscarriage from viable intrauterine pregnancy. Fertil Steril.

[R155] Sun Y, Tian Z, Zhao H, Wong ST, Chen B (2007). Characteristic of hypothalamic kisspeptin expression in the pubertal development of precocious female rats. Neurosci Lett.

[R156] Takayanagi Y, Onaka T (2010). Roles of prolactin-releasing peptide and RFamide related peptides in the control of stress and food intake. FEBS J.

[R157] Talbot F, Feetham CH, Mokrosiński J, Lawler K, Keogh JM, Henning E, Mendes de Oliveira E, Ayinampudi V, Saeed S, Bonnefond A, Arslan M, Yeo GSH, Froguel P, Bechtold DA, Adamson A, Humphreys N, Barroso I, Luckman SM, Farooqi IS (2023). A rare human variant that disrupts GPR10 signalling causes weight gain in mice. Nat Commun.

[R158] Tanaka A, Nakata D, Masaki T, Kusaka M, Watanabe T, Matsui H (2018). Evaluation of pharmacokinetics/pharmacodynamics and efficacy of one-month depots of TAK-448 and TAK-683, investigational kisspeptin analogs, in male rats and an androgen-dependent prostate cancer model. Eur J Pharmacol.

[R159] Timofeev I, Chauvette S (2011). Thalamocortical oscillations: local control of EEG slow waves. Curr Top Med Chem.

[R160] Trevisan CM, Montagna E, de Oliveira R, Christofolini DM, Barbosa CP, Crandall KA, Bianco B (2018). Kisspeptin/GPR54 system: what do we know about its role in human reproduction?. Cell Physiol Biochem.

[R161] Tsoutsouki J, Patel B, Comninos AN, Dhillo WS, Abbara A (2022). Kisspeptin in the Prediction of Pregnancy Complications. Front Endocrinol (Lausanne).

[R162] Ubuka T, Son YL, Bentley GE, Millar RP, Tsutsui K (2013). Gonadotropin-inhibitory hormone (GnIH), GnIH receptor and cell signaling. Gen Comp Endocrinol.

[R163] Ukena K, Tachibana T, Iwakoshi-Ukena E, Saito Y, Minakata H, Kawaguchi R, Osugi T, Tobari Y, Leprince J, Vaudry H, Tsutsui K (2010). Identification, localization, and function of a novel avian hypothalamic neuropeptide, 26RFa, and its cognate receptor, G protein-coupled receptor-103. Endocrinology.

[R164] Vas S (2023). Prolactin-releasing peptide contributes to stress-related mood disorders and inhibits sleep/mood regulatory melanin-concentrating hormone neurons in rats. J Neurosci.

[R165] Wang X, Song K, Li L, Chen L (2018). Structure-based drug design strategies and challenges. Curr Top Med Chem.

[R166] Wang ZL, Li N, Wang P, Tang HH, Han ZL, Song JJ, Li XH, Yu HP, Zhang T, Zhang R, Xu B, Zhang MN, Fang Q, Wang R (2016). Pharmacological characterization of EN-9, a novel chimeric peptide of endomorphin-2 and neuropeptide FF that produces potent antinociceptive activity and limited tolerance. Neuropharmacology.

[R167] Waqas SFH (2017). Neuropeptide FF increases M2 activation and self-renewal of adipose tissue macrophages. J Clin Invest.

[R168] Wei H, Panula P, Pertovaara A (2001). Modulation of pain by [1DMe] NPYF, a stable analogue of neuropeptide FF, in neuropathic rats. Brain Res.

[R169] Wickramasuriya N, Hawkins R, Atwood C, Butler T (2022). The roles of GnRH in the human central nervous system. Horm Behav.

[R170] Wold EA, Chen J, Cunningham KA, Zhou J (2019). Allosteric modulation of class A GPCRs: targets, agents, and emerging concepts. J Med Chem.

[R171] Wu Z, Jia Q, Liu B, Fang L, Leung PCK, Cheng JC (2023). NPFF stimulates human ovarian cancer cell invasion by upregulating MMP-9 via ERK1/2 signaling. Exp Cell Res.

[R172] Wu Z, Chen G, Qiu C, Yan X, Xu L, Jiang S, Xu J, Han R, Shi T, Liu Y, Gao W, Wang Q, Li J, Ye F, Pan X, Zhang Z, Ning P, Zhang B, Chen J, Du Y (2024). Structural basis for the ligand recognition and G protein subtype selectivity of kisspeptin receptor. Sci Adv.

[R173] Xu B, Guo Y, Zhang M, Zhang R, Chen D, Zhang Q, Xiao J, Xu K, Li N, Qiu Y, Zhu H, Niu J, Zhang X, Fang Q (2020). Central and peripheral modulation of gastrointestinal transit in mice by DN-9, a multifunctional opioid/NPFF receptor agonist. Neurogastroenterol Motil.

[R174] Yakovlev OA, Vengerovich NG, Nikiforov AS, Vakhviyaynen MS (2022). Pharmacological potential of ligands to receptors of RF-amide neuropeptide system. Pharm Formulas.

[R175] Yang HY, Tao T, Iadarola MJ (2008). Modulatory role of neuropeptide FF system in nociception and opiate analgesia. Neuropeptides.

[R176] Yu HP, Zhang N, Zhang T, Wang ZL, Li N, Tang HH, Zhang R, Zhang MN, Xu B, Fang Q, Wang R (2016). Activation of NPFF2 receptor stimulates neurite outgrowth in Neuro 2A cells through activation of ERK signaling pathway. Peptides.

[R177] Zeydabadi Nejad S, Ramezani Tehrani F, Zadeh-Vakili A (2017). The role of kisspeptin in female reproduction. Int J Endocrinol Metab.

[R178] Zhang C, Rønnekleiv OK, Kelly MJ (2013). Kisspeptin inhibits a slow afterhyperpolarization current via protein kinase C and reduces spike frequency adaptation in GnRH neurons. Am J Physiol Endocrinol Metab.

[R179] Zhang L, Koller J, Gopalasingam G, Herzog H (2022). NPFF signalling is critical for thermosensory and dietary regulation of thermogenesis. Neuropeptides.

[R180] Zhang S, Yu F, Che A, Tan B, Huang C, Chen Y, Liu X, Huang Q, Zhang W, Ma C, Qian M, Liu M, Qin J, Du B (2022). Neuroendocrine regulation of stress-induced T cell dysfunction during lung cancer immunosurveillance via the Kisspeptin/GPR54 signaling pathway. Adv Sci (Weinh).

[R181] Zhang SQ, Kimura M, Inoué S (2000). Effects of prolactin-releasing peptide (PrRP) on sleep regulation in rats. Psychiatry Clin Neurosci.

[R182] Zhang SQ, Inoué S, Kimura M (2001). Sleep-promoting activity of prolactin-releasing peptide (PrRP) in the rat. Neuroreport.

[R183] Zhang T, Zhao W, Zhang M, Xu B, Shi X, Zhang Q, Guo Y, Xiao J, Chen D, Zheng T, Fang Q (2018). Analgesic activities of the mixed opioid and NPFF receptors agonist DN-9 in a mouse model of formalin-induced orofacial inflammatory pain. Peptides.

[R184] Zhu LL, Onaka T (2003). Facilitative role of prolactin-releasing peptide neurons in oxytocin cell activation after conditioned-fear stimuli. Neuroscience.

